# VCAM-1 Promotes Angiogenesis of Bone Marrow Mesenchymal Stem Cells Derived from Patients with Trauma-Induced Osteonecrosis of the Femoral Head by Regulating the Apelin/CCN2 Pathway

**DOI:** 10.1155/2023/6684617

**Published:** 2023-10-12

**Authors:** Yiming Shao, Lei Sun, Baodong Ma, Ranran Jin, Yueyao Ban, Ruibo Li, Jianfa Wang, Hongkai Lian, Han Yue

**Affiliations:** ^1^Department of Orthopedics, Zhengzhou Central Hospital Affiliated to Zhengzhou University, Zhengzhou 450001, China; ^2^Center of Stem Cell and Regenerative Medicine, Zhengzhou Central Hospital Affiliated to Zhengzhou University, Zhengzhou 450001, China; ^3^Department of Trauma Surgery, Zhengzhou Central Hospital Affiliated to Zhengzhou University, Zhengzhou 450001, China

## Abstract

Trauma-induced osteonecrosis of the femoral head (TI-ONFH) is a pathological process in which the destruction of blood vessels supplying blood to the femoral head causes the death of bone tissue cells. Vascular cell adhesion molecule 1 (VCAM-1) has been shown to have potent proangiogenic activity, but the role in angiogenesis of TI-ONFH is unclear. In this work, we discovered that VCAM-1 was significantly downregulated in the bone marrow mesenchymal stem cells (BMSCs) derived from patients with TI-ONFH. Subsequently, we constructed BMSCs overexpressing VCAM-1 using a lentiviral vector. VCAM-1 enhances the migration and angiogenesis of BMSCs. We further performed mRNA transcriptome sequencing to explore the mechanisms by which VCAM-1 promotes angiogenesis. Gene ontology biological process enrichment analysis demonstrated that upregulated differentially expressed genes (DEGs) were related to blood vessel development. Kyoto Encyclopedia of Genes and Genomes pathway enrichment analysis revealed that upregulated DEGs were engaged in the Apelin signaling pathway. Apelin-13 is the endogenous ligand of the APJ receptor and activates this G protein-coupled receptor. Treatment with Apelin-13 activated the Apelin signaling pathway and suppressed the expression of cellular communication network factor 2 in BMSCs. Furthermore, Apelin-13 also inhibits the migration and angiogenesis of VCAM-1-BMSCs. In summary, VCAM-1 plays an important role in vascular microcirculation disorders of TI-ONFH, which provides a new direction for the molecular mechanism and treatment of TI-ONFH.

## 1. Introduction

Osteonecrosis of the femoral head (ONFH) is a pathological process of bone tissue necrosis usually caused by hip trauma, alcoholism, and long-term administration of steroids [1]. This can progress to bone collapse and secondary hip osteoarthritis, causing severe hip pain and loss of joint function. For patients with advanced ONFH, total hip arthroplasty is currently the best option [2]. However, hip preservation therapy remains a challenge in young adults or active populations. The currently accepted etiological theory of trauma-induced ONFH (TI-ONFH) is that hip trauma leads to local vascular disruption, causing vascular microcirculation disorders and leading to osteonecrosis [3]. However, the molecular mechanisms associated with impaired vascular microcirculation in TI-ONFH are still unclear.

Numerous studies have shown that mesenchymal stem cells (MSCs) from different tissue sources exhibit excellent angiogenic effects [4–6]. A number of angiogenic factors and enzymes secreted by MSCs have been widely reported to initiate angiogenesis. Bone marrow MSCs (BMSCs) are capable of interacting with endothelial progenitor cells to enhance angiogenesis by promoting the secretion of vascular endothelial growth factor (VEGF) and platelet-derived growth factor [7]. BMSCs also strengthened the in vitro angiogenic sprouting capacity of human umbilical vein endothelial cells (HUVECs) in a hepatocyte growth factor-dependent manner [8].

Vascular cell adhesion molecule 1 (VCAM-1), also known as CD106, is a 110 kDa glycoprotein that is inducible and predominantly expressed in endothelial cells [9]. In the inflammatory response, VCAM-1 acts as a cell adhesion molecule by directly interacting with *α*4*β*1 integrin expressed on leukocytes via the Ig-like domains 1 and 4 within the extracellular domain [10]. In addition, soluble VCAM-1 (sVCAM-1) has been shown to mediate angiogenesis in the rat cornea [11], and the sVCAM-1/*α*4 integrin pathway plays an important role in inflammatory stimuli-induced angiogenesis [12, 13]. Recent studies demonstrated that VCAM-1 in placenta chorionic villi-derived MSCs shows potent proangiogenic activity [14]. However, the role of VCAM-1 in TI-ONFH-derived BMSCs has not been explored.

**Figure 1:**
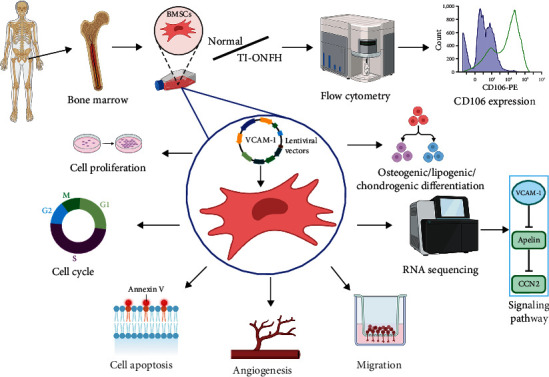
The schematic diagram of this study.

Therefore, the aim of this study was to verify the expression and the ability to promote the angiogenesis of VCAM-1 in BMSCs derived from TI-ONFH patients. In addition, we performed RNA sequencing and further validation to reveal the possible mechanisms by which VCAM-1 promotes angiogenesis. The protocol of our experiment is shown in Figure 1.

## 2. Materials and Methods

### 2.1. Clinical Sample Data

Patients who developed ONFH after previous treatment for hip trauma were included in this study. All patients underwent bilateral hip joint X-ray and magnetic resonance imaging (MRI) examination. Patients were not receiving any medication within the past 6 months and had no other joint disease, autoimmune disease, systemic inflammation, malignant, or chronic disease. In addition, patients with simple femoral neck fractures were included in the control group.

This study was approved by the Ethical Committee of the Zhengzhou Central Hospital Affiliated to Zhengzhou University (Ethics number: 202170). All volunteers have signed informed consent.

### 2.2. Cell Isolation and Culture

Bone marrow was collected from patients, and BMSCs were extracted. The regular culture medium for BMSCs was MSC serum-free media (Yocon, China) and MSC serum-free media additive (Yocon, China). Cells were cultured at 37°C with 5% CO_2_ in a humidified environment. HUVECs were cultured in a high-glucose DMEM medium (Procell, China). BMSCs and HUVECs were cocultured using transwell chambers (Corning, USA).

To investigate the role of the Apelin signaling pathway in the effects of VCAM-1, BMSCs were treated with 1 nM Apelin-13 (Cayman Chemical, USA) for 2 hr after reaching 80%–90% confluence [15].

### 2.3. Flow Cytometry Analysis

The phenotype of BMSCs was analyzed using the following antibodies: phycoerythrin-conjugated CD90 and CD106; allophycocyanin-conjugated CD73 and CD105, and fluorescein isothiocyanate-conjugated CD14, CD19, CD34, CD45, and human leukocyte antigens-DR (HLA-DR). Cells were examined by CytoFLEX flow cytometer (Beckman Coulter, USA). All of the antibodies were purchased from Biolegend (USA), and the flow cytometry data were analyzed by CytExpert software (Beckman Coulter, USA).

### 2.4. Osteogenic, Lipogenic, and Chondrogenic Differentiation

BMSCs were trypsinized and seeded in a 24-well plate at a concentration of 2 × 10^4^ cells per well. BMSCs were induced using osteogenic and lipogenic media when reaching 80%–90% confluence. The induction medium was changed every 3 days. After 14 days of induction, mineral deposition was detected using Alizarin Red S staining (Beyotime, China), and lipid droplets were detected using Oil Red O staining (Solarbio, China).

BMSCs (5 × 10^5^ cells) were centrifuged at 1,500 rpm for 5 min to obtain cell pellets. The cell pellets were cultured in chondrogenic medium for up to 21 days. Cells were fixed in 4% formaldehyde, dehydrated in an ethanol series, and embedded in paraffin blocks. Blocks were cut, and sections were stained with Alcian Blue (Procell, China) to evaluate chondrogenic differentiation.

### 2.5. Lentiviral Vector Transduction

Lentiviral vectors carrying genes for VCAM-1 and control vectors were prepared by Jikai Gene (China). Cell suspension at a density of 2.5 × 10^5^/ml was prepared in MSC serum-free medium and seeded at 5 × 10^5^ cells per well onto 6-well plates. Premixed virus vector (15 *μ*l) with 40 *μ*l HiTransG A transfection agent (Jikai Gene). The culture medium was changed after 16 hr transfection with OPTI-MEM. After 48 hr, cells were observed under a fluorescent microscope for transfection. The successful-transduced cells were screened with puromycin.

### 2.6. Quantitative Real-Time Polymerase Chain Reaction (qRT-PCR)

Total RNA was extracted from BMSCs by using the FastPure® Cell/Tissue Total RNA Isolation Kit V2 (Vazyme, China). Next, cDNA was synthesized from 1 *μ*g of total RNA by using the RevertAid First Strand cDNA Synthesis Kit (Thermo Scientific, USA). Then, qRT-PCR analysis was performed with ChamQ Universal SYBR qPCR Master Mix (Vazyme, China) in the 7500 Fast Dx Real-Time PCR System (Applied Biosystems, USA). The relative standard curve method (2^−*ΔΔ*CT^) was used to determine the relative RNA expression, using *β*-actin as the reference. The PCR primers used in this study are shown in Table 1.

### 2.7. Western Blotting

Protein extracts were separated by sodium dodecyl sulfate-polyacrylamide gel electrophoresis and transferred to polyvinylidene fluoride membranes. Blots were blocked with 5% milk in Tris-buffered saline containing 0.5% Tween-20 for 1 hr at room temperature. The membranes were incubated with primary antibodies at 4°C overnight, followed by incubation with the horseradish peroxidase-conjugated secondary antibodies at 37°C for 2 hr. The immunoreactive bands were visualized using Omni-ECL™ Femto Light Chemiluminescence Kit (Epizyme, China) and imaged by the ChemiDoc XRS Plus luminescent image analyzer (Bio-Rad, USA). The antibodies used in this study were as follows: anti-glyceraldehyde-3-phosphate dehydrogenase (1 : 5,000, Bioworld Technology, USA), anti-VCAM-1 (1 : 1,000, Cell Signaling Technology, USA), anti-VEGF (1 : 1,000, Abcam, UK), anti-PLGF (1 : 1,000, Abcam, UK), anti-FGF2 (1 : 1,000, Abcam, UK), anti-APLN (1 : 1,000, Abcam, UK), anti-cellular communication network factor 2 (CCN2) (1 : 1,000, Abcam, UK), horseradish peroxidase conjugated anti-rabbit IgG (1 : 5,000, Abcam, UK).

### 2.8. Evaluation of Apoptosis

BMSCs were digested, washed, suspended with Annexin V binding buffer, and counted to ensure at least 1 × 10^5^ cells in a test. The cell suspension was incubated with Annexin V-AbFluor 647 and propidium iodide (PI). Then kept away from light for 15 min, and apoptosis was detected by flow cytometry. The apoptosis detection kit was purchased from Abbkine (China).

### 2.9. Proliferation Assay

The proliferation of BMSCs was detected by cell counting kit-8 (CCK-8; Epizyme, China). Briefly, 2 × 10^3^ cells/well (three replicates per group) were seeded into 96-well plates and cultured at 37°C with 5% CO_2_ for 1, 3, 5, 7, and 9 days. CCK-8 solution (10 *μ*l) was added to each well and incubated at 37°C for 2 hr. The absorbance was observed at 450 nm by using a microplate reader (Molecular Devices, USA).

### 2.10. Cell Cycle

BMSCs were fixed in 70% ethanol (4°C) overnight. Then, the fixed cells were washed with PBS and incubated in RNase A (7 sea, China) and PI for 30 min. The distribution of the cell cycle (G0/G1, S, and G2/M) was detected using a flow cytometer.

### 2.11. Transwell

Migration of BMSCs was assessed in a 24-well plate using transwell chambers with 8 *μ*m pore size (Corning, USA). MSCs were suspended in a serum-free medium, and 100 *μ*l (1 × 10^6^/ml) of the cell suspension was added to the upper chamber of the migration wells. Then, 600 *μ*l of DMEM medium containing 10% FBS was added to each lower chamber. After 24 hr, BMSCs passing through the upper chamber membrane were fixed with 4% paraformaldehyde (Solarbio, China) and stained with 1% crystal violet dye solution (Solarbio, China). Micrographs were taken for each chamber, and the cell numbers (three replicate readings per group) were counted manually.

### 2.12. Tube Formation Assay

Matrigel (150 *µ*l) (Corning, USA) was added to each well of a 48-well plate and allowed to polymerize. HUVECs and BMSCs were mixed (1 × 10^5^ cells, 1 : 1) and plated on Matrigel. After culture for 6 hr, images were taken using a microscope. The tube formation was quantified by analyzing the total tube length in each well with ImageJ (National Institutes of Health, USA).

### 2.13. RNA-Sequence and Bioinformatics Analysis

Three samples from each group were analyzed by RNA sequencing. Differentially expressed genes (DEGs) were identified based on the criteria of |log2 (fold change)| ≥1 and FDR ≤ 0.05 and then visualized as volcano and heat maps. All DEGs were submitted to the Metascape (https://metascape.org/) database for gene ontology (GO) function enrichment analysis and Kyoto Encyclopedia of Genes and Genomes (KEGG) pathway enrichment analysis and are shown as bubble plots.

### 2.14. Statistical Analysis

Data were expressed as mean ± standard deviation. A two-tailed unpaired Student's *t*-test was performed for comparison between two groups. All analyses were conducted with GraphPad Prism 8. *P* < 0.05 was considered a significant difference.

## 3. Results

### 3.1. Identification and Characterization of BMSCs

**Figure 2:**
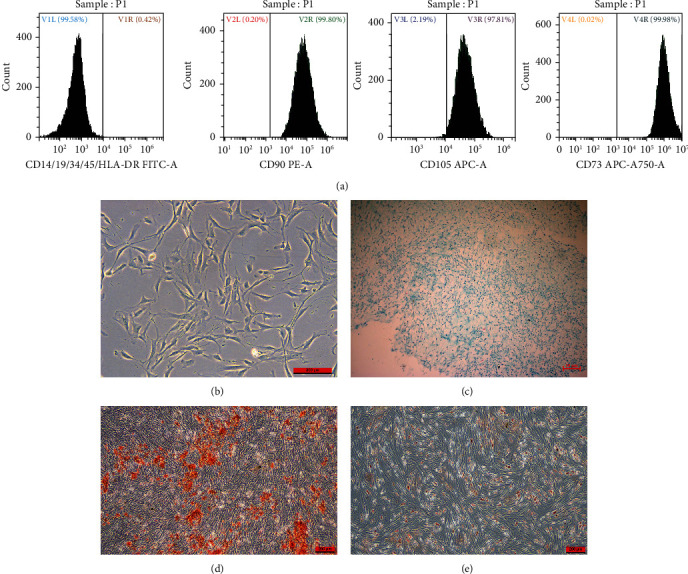
The characterization of BMSCs: (a) BMSCs were positive for CD73, CD90, and CD105, while negative for CD14, CD19, CD34, CD45, and HLA-DR; (b) morphology of isolated undifferentiated BMSCs; (c) Alcian blue staining for chondrogenic differentiation of BMSCs; (d) Alizarin red S staining for osteogenic differentiation of BMSCs; (e) oil red O staining for lipid differentiation of BMSCs.

The results of flow cytometry showed that the BMSCs derived from patients were positive for CD73, CD90, and CD105 but negative for CD14, CD19, CD34, CD45, and HLA-DR (Figure 2(a)). BMSCs without the addition of induction an medium were adherent and arranged radially (Figure 2(b)). Blue-stained acid proteoglycan was observed by Alcian Blue staining (Figure 2(c)); matrix mineralization was observed with Alizarin Red S staining (Figure 2(d)); lipid droplet formation was observed with Oil Red O staining (Figure 2(e)). The above results are consistent with the characteristics of MSCs.

### 3.2. VCAM-1 Was Significantly Downregulated in the BMSCs Derived from Patients with TI-ONFH

**Figure 3:**
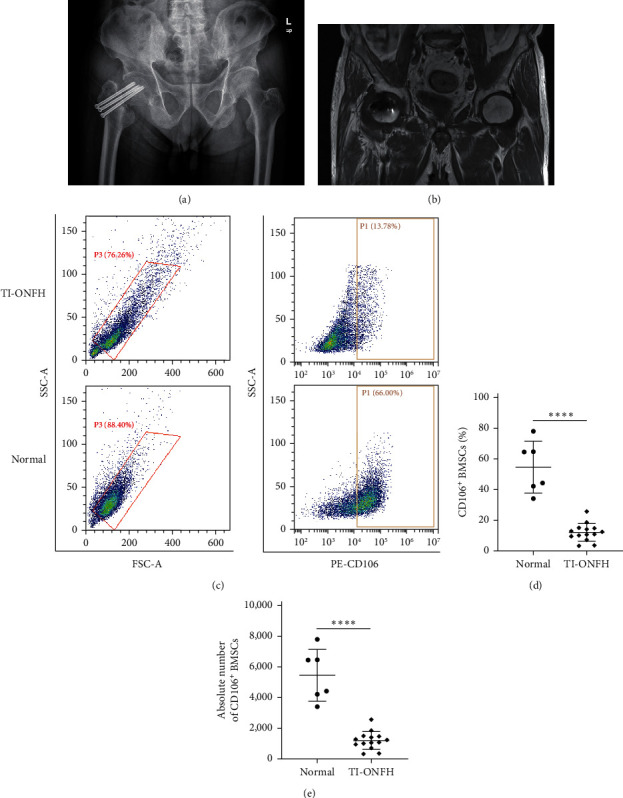
Expression of VCAM-1 in BMSCs of patients with TI-ONFH: (a) typical X-ray image of a patient with TI-ONFH; (b) typical MRI image of a patient with TI-ONFH; (c) CD106 expression was detected by flow cytometry; (d) percentage of CD106 in BMSCs; (e) absolute number of CD106 in BMSCs.  ^*∗*^ ^*∗*^ ^*∗*^ ^*∗*^*P* < 0.0001.

Fourteen patients who developed ONFH after treatment for hip trauma were included in the experimental group (Figures 3(a) and 3(b)). In the same period, six patients with simple femoral neck fractures were included in the control group. Considering the effect of aging on MSCs, there was no statistical difference between the age of patients in the experimental group (59–71, 64.86 ± 3.66) and the control group (65–70, 67.33 ± 2.07). Additionally, there was no significant difference between the gender of the patients in the experimental group (eight males and six females) and the control group (two males and four females). The information of all patients is shown in Table 2. To explore the potential role of VCAM-1 in TI-ONFH, we detected the expression of CD106 in BMSCs by flow cytometry (Figure 3(c)). The results revealed that CD106 was significantly downregulated in BMSCs from patients with TI-ONFH (Figures 3(d) and 3(e)).

### 3.3. Overexpression of VCAM-1 Did Not Alter the Properties of BMSCs

**Figure 4:**
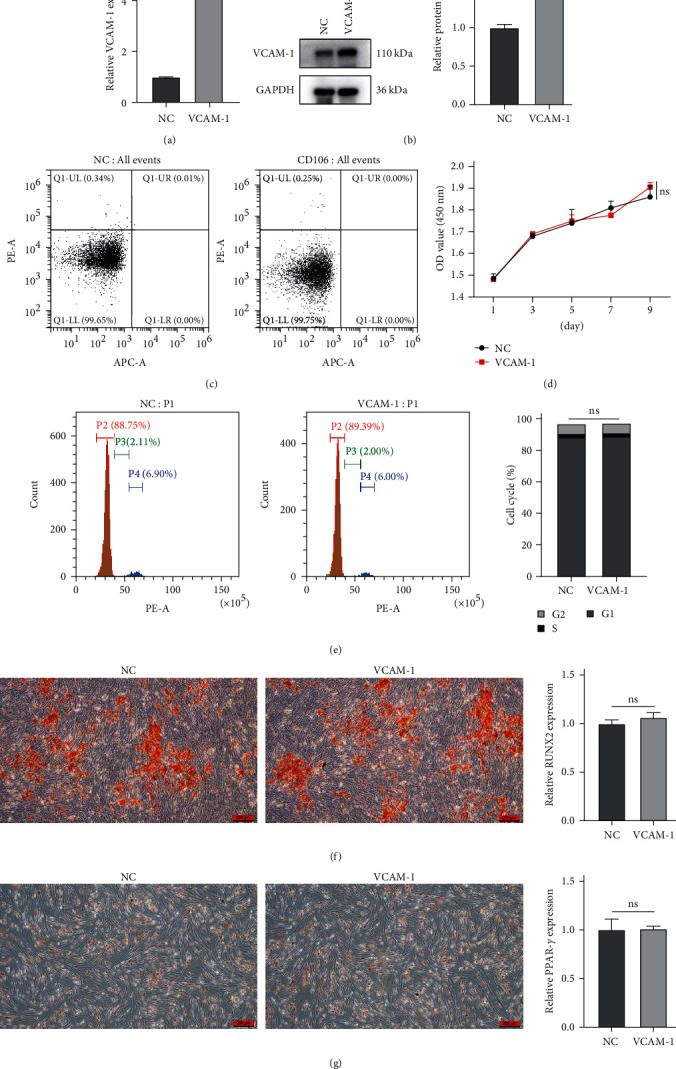
Effects of VCAM-1 on the properties of BMSCs: (a) the relative mRNA expression of VCAM-1; (b) the relative protein expression of VCAM-1; (c) cell apoptosis; (d) cell proliferation; (e) cell cycle; (f) osteogenic differentiation and the relative mRNA expression of RUNX2; (g) lipogenic differentiation and the relative mRNA expression of PPAR*γ*.  ^*∗*^ ^*∗*^ ^*∗*^*P* < 0.001;  ^*∗*^ ^*∗*^ ^*∗*^ ^*∗*^*P* < 0.0001; ns, no significance.

After lentiviral transfection of BMSCs, Western blot and qRT-PCR verified the expression of VCAM-1 (Figures 4(a) and 4(b)). Flow cytometry results showed that overexpression of VCAM-1 did not affect the apoptosis of BMSCs (Figure 4(c)). CCK-8 assay confirmed that there was no significant difference in cell proliferation between the two groups of BMSCs (Figure 4(d)). In addition, VCAM-1 had no effect on the cell cycle of BMSCs (Figure 4(e)). Finally, we wanted to know whether VCAM-1 could directly influence the multidirectional differentiation potential of BMSCs. However, the osteogenic and lipogenic differentiation of BMSCs did not differ significantly (Figures 4(f) and 4(g)).

### 3.4. Overexpression of VCAM-1 Enhanced Migration and Angiogenesis of BMSCs

**Figure 5:**
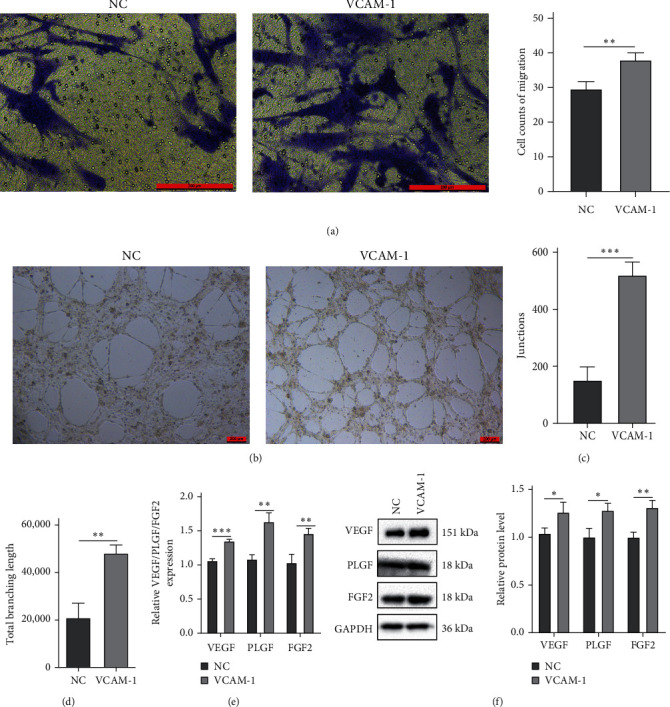
VCAM-1 enhanced migration and angiogenesis of BMSCs: (a) cell counts of migration; (b) typical image of tube formation; (c) junctions of angiogenic network; (d) total branching length of angiogenic network; (e) the relative mRNA expression of VEGF, PLGF, and FGF2 in HUVECs; (f) the relative protein expression of VEGF, PLGF, and FGF2 in HUVECs.

The migration capacity of BMSCs was assessed by the transwell chamber. After 24 hr of incubation, more BMSCs crossed the upper chamber membrane in the VCAM1 group (Figure 5(a)). The angiogenic capability of BMSCs was assessed using an in vitro capillary-like structure (tube) formation assay on the basement membrane matrix. Tube formation peaked after 6 hr of coculture between 1 : 1 mixture of BMSCs and HUVEC (Figure 5(b)). We analyzed the parameters in the angiogenic network and found that the VCAM-1 group formed more junctions and total branch length (Figures 5(c) and 5(d)). After the coculture of BMSCs and HUVECs, the expression of angiogenesis-related factors (VEGF, PLGF, and FGF2) was significantly upregulated in HUVECs (Figures 5(e) and 5(f)).

### 3.5. Bioinformatics Analyses

**Figure 6:**
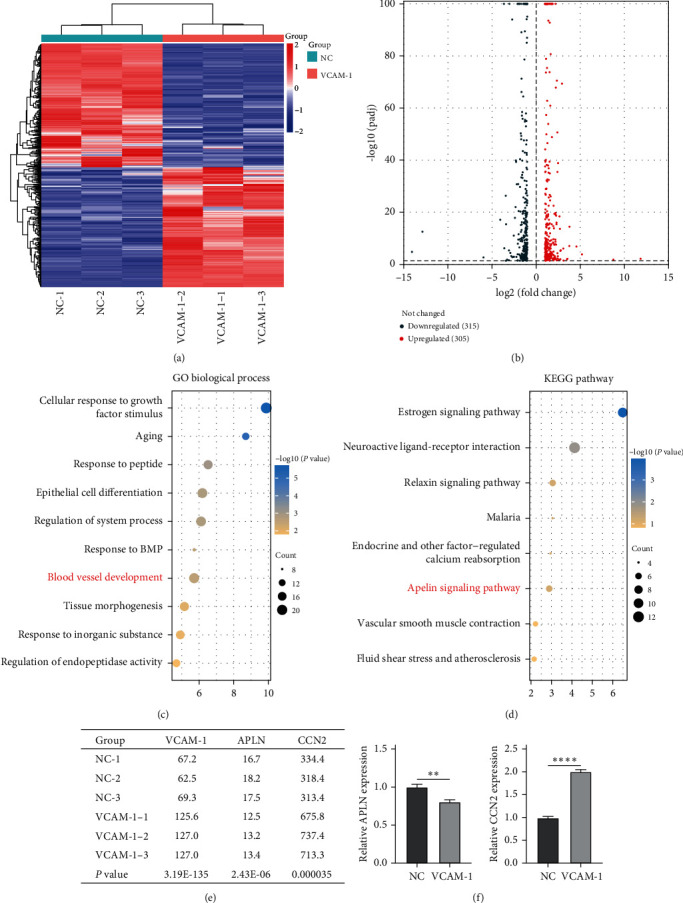
Bioinformatics analyses: (a) heat map of DEGs; (b) volcano map of DEGs; (c) GO biological process of upregulating DEGs; (d) KEGG pathway enrichment of upregulating DEGs; (e) gene expression of VCAM-1, APLN, and CCN2 (TPM); (f) the relative mRNA expression of APLN and CCN2. TPM, transcripts per million.

After differential expression analysis of the raw sequencing data, 305 upregulated genes and 315 downregulated genes were obtained (Figures 6(a) and 6(b); Supplementary 1). GO biological process enrichment analysis revealed that upregulated DEGs were involved in blood vessel development and cellular response to growth factor stimulus (Figure 6(c)). KEGG pathway enrichment analysis showed that upregulated DEGs were involved in the Apelin signaling pathway, estrogen signaling pathway, and relaxin signaling pathway (Figure 6(d)). RNA-seq results showed that overexpression of VCAM-1 resulted in the downregulation of APLN (gene for apelin) and upregulation of CCN2 (Figure 6(e)). We verified the above results using qRT-PCR (Figure 6(f)).

### 3.6. Apelin-13 Inhibits the Migration and Angiogenesis of VCAM-1-BMSCs

**Figure 7:**
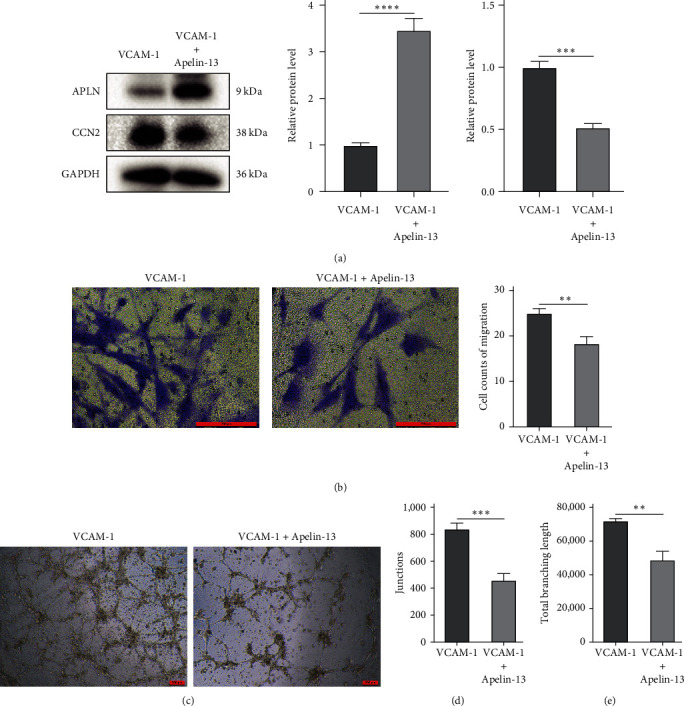
Apelin-13 inhibits the migration and angiogenesis of VCAM-1-BMSCs: (a) the relative protein expression of APLN and CCN2 after treatment with Apelin-13; (b) cell counts of migration after treatment with Apelin-13; (c) typical image of tube formation after treatment with Apelin-13; (d) junctions of angiogenic network after treatment with Apelin-13; (e) total branching length of angiogenic network after treatment with Apelin-13.

Apelin-13 is the predominant subtype of Apelin found in the heart and brain. Apelin-13 is the endogenous ligand of the APJ receptor and activates this G protein-coupled receptor. To validate the results of bioinformatic analysis, we used Apelin-13 to activate APLN and inhibit the expression of CCN2 in BMSCs (Figure 7(a)). In addition, the number of BMSCs crossing the upper chamber membrane became less (Figure 7(b)). More importantly, Apelin-13 inhibited the angiogenic capacity of BMSCs (Figure 7(c)). Both the junctions and total branch length in the angiogenic network were significantly reduced (Figures 7(d) and 7(e)).

## 4. Discussion

**Figure 8:**
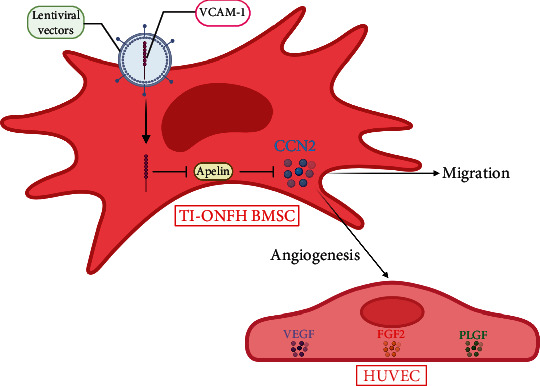
VCAM-1 promotes angiogenesis and migration of BMSCs by regulating the Apelin/CCN2 pathway.

Previous studies have reported that VCAM-1 in placenta chorionic villi-derived MSCs exhibits excellent angiogenic paracrine activity and displayed therapeutic efficacy on mouse hindlimb ischemia [14]. This study evidenced the angiogenic potential of VCAM-1 in BMSCs from patients with TI-ONFH. In addition, we further demonstrated that VCAM-1 promotes angiogenesis and migration of BMSCs by regulating the Apelin/CCN2 pathway (Figure 8).

VCAM-1, as an adhesion molecule within the vasculature, has been shown to be involved in the angiogenic process in a variety of diseases. VCAM-1 was released into the blood as sVCAM-1 after cleavage from the cell surface, and serum sVCAM-1 levels were significantly higher in gastric cancer patients than in normal subjects [16]. Sano et al. [17] detected high expression of VCAM-1 in thrombi formed in pancreatic ductal adenocarcinoma and significantly inhibited tumor angiogenesis after intervention with anti-VCAM-1 antibodies.

In view of the importance of angiogenesis in ONFH, we investigated the expression of VCAM-1 in BMSCs from patients with ONFH due to different etiologies. In the present study, we found that VCAM-1 was significantly downregulated in BMSCs from patients with TI-ONFH. In the tube formation assay, overexpression of VCAM-1 in BMSCs enhanced the tube formation of HUVECs. It implicates that VCAM-1 plays an important role in vascular microcirculation disorders in TI-ONFH.

To further explore the mechanism by which VCAM-1 promotes angiogenesis, we performed RNA sequencing analysis. The results of GO biological process enrichment analysis showed that upregulated DEGs are involved in blood vessel development. Furthermore, the results of KEGG enrichment analysis showed that upregulated DEGs are involved in the apelin signaling pathway. Apelin is a vasoactive peptide and is an endogenous ligand for APJ receptors, which are widely expressed in blood vessels, heart, and cardiovascular regulatory regions of the brain [18]. Apelin and APJ are also expressed on ECs of the newly developing blood vessels and mediate angiogenesis [19]. It has been reported that Apelin deficiency significantly impaired retinal vascularization in the early postnatal period [20]. In addition, in the hind limb ischemia model, apelin, together with VEGF, effectively induced functional vessels larger than with VEGF alone [21]. This may be caused by the Apelin/APJ system that induces the proliferation and migration of ECs and drives vascular endothelial cells toward a proangiogenic state [22, 23]. Interestingly, in this study, overexpression of VCAM-1 downregulated the expression of APLN. This suggests that the angiogenic function played by VCAM-1 in BMSCs may not be exerted directly by apelin.

CCN2, also known as connective tissue growth factor, is a cysteine-rich matricellular protein involved in the regulation of a variety of biological processes [24]. In this study, we found that overexpression of VCAM-1 upregulated the expression of CCN2. Then, administration of apelin-13 downregulated the expression of CCN2 and inhibited the angiogenesis and migration of BMSCs. Previously, the Apelin/CCN2 pathway was known for its ability to modulate fibrosis [25]. We identified the role of the apelin/CCN2 axis in angiogenesis for the first time. CCN2 has been shown to induce angiogenesis and to promote adhesion, migration, and survival of ECs [26]. This further confirms our view that overexpression of VCAM-1 promotes BMSCs migration and angiogenesis via Apelin/CCN2.

However, the present study has the following limitations. First, the angiogenic ability of VCAM-1 gene-modified BMSCs needs to be further validated with in vivo experiments. Second, because of the powerful proangiogenic function of VCAM-1, its additional mechanisms and role in the bone marrow microenvironment remain to be explored.

## 5. Conclusion

Taken together, the present study revealed a significant downregulation of VCAM-1 in BMSCs from patients with TI-ONFH. Furthermore, VCAM-1 promoted the migration and angiogenic capacity of BMSCs through the Apelin/CCN2 signaling pathway. Our findings provide new insights into the molecular mechanisms of TI-ONFH progression.

## Figures and Tables

**Table 1:** Primer sequences.

Gene		Sequence
VCAM-1	Forward	GGACCACATCTACGCTGACA
	Reverse	TTGACTGTGATCGGCTTCCC
RUNX2	Forward	AGGCAGTTCCCAAGCATTTCATCC
	Reverse	TGGCAGGTAGGTGTGGTAGTGAG
PPAR*γ*	Forward	AGATCATTTACACAATGCTGGC
	Reverse	TAAAGTCACCAAAAGGCTTTCG
VEGF	Forward	AAGATCCGCAGACGTGTAAATGTT
	Reverse	CCCCAAAAGCAGGTCACTCAC
PLGF	Forward	TGTCACCATGCAGCTCCTAA
	Reverse	CCGGCATTCGCAGCGAACGTGC
FGF2	Forward	CTGGCTATGAAGGAAGATGGA
	Reverse	TGCCCAGTTCGTTTCAGTG
APLN	Forward	TGCTCTGGCTCTCCTTGAC
	Reverse	CTGGAGGTCTGCGAGGAACA
CCN2	Forward	TGGCATGAAGCCAGAGAGTG
	Reverse	GTGGGAATCTTTTCCCCCAGT
*β*-actin	Forward	TCACCATGGATGATGATATCGC
	Reverse	ATAGGAATCCTTCTGACCCATGC

**Table 2:** Patient information.

Group	Age	Gender	Disease	Side
Experimental	65	F	TI-ONFH	R
Experimental	71	M	TI-ONFH	L
Experimental	63	M	TI-ONFH	L
Experimental	64	F	TI-ONFH	R
Experimental	63	F	TI-ONFH	R
Experimental	66	F	TI-ONFH	R
Experimental	59	M	TI-ONFH	L
Experimental	65	M	TI-ONFH	R
Experimental	65	M	TI-ONFH	L
Experimental	61	M	TI-ONFH	R
Experimental	66	F	TI-ONFH	L
Experimental	70	M	TI-ONFH	R
Experimental	60	F	TI-ONFH	R
Experimental	70	M	TI-ONFH	R
Control	69	M	FNF	R
Control	68	F	FNF	L
Control	67	F	FNF	L
Control	65	F	FNF	L
Control	65	M	FNF	R
Control	70	F	FNF	L

TI-ONFH, trauma induced-osteonecrosis of the femoral head; FNF, femoral neck fracture.

## Data Availability

The data used to support the findings of this study are included within the article and the supplementary information file.

## Supplementary Materials

Supplementary Materials Supplementary 1. Results of differential expression analysis.
