# Supplementary material for: VCAM-1 Promotes Angiogenesis of Bone Marrow Mesenchymal Stem Cells Derived from Patients with Trauma-Induced Osteonecrosis of the Femoral Head by Regulating the Apelin/CCN2 Pathway
Source: Stem Cells Int. 2023 Oct 12;2023:6684617. doi: 10.1155/2023/6684617 (PMC10586908; doi:10.1155/2023/6684617)
Supplement: Supplementary Materials — Supplementary 1. Results of differential expression analysis. [file 6684617.f1.pdf]

Differential expression analysis

GeneSymbol	log2FC	pvalue	NC_1	NC_2	NC_3	CD106_1	CD106_2	CD106_3
LGMN	1.154464	1.68E-255	104.8184	105.0513	105.5073	218.9602	215.5103	219.3279
GPR183	1.310479	0.000507	1.36148	0.647405	0.666628	2.601913	2.161401	1.567689
MARCHF4	1.274818	6.49E-49	5.032882	5.814203	4.903289	12.32844	11.99533	12.04817
KRT33B	1.611884	9.96E-05	0.498257	0.46348	0.37967	1.427082	1.447517	1.077385
ACTL10	1.343928	8.26E-05	1.877156	1.259315	0.702059	2.968646	3.241404	2.812845
GCLC	1.120956	1.24E-81	16.65035	15.6136	17.35627	33.88926	33.56014	35.08963
PLPP4	2.016344	1.42E-15	0.415186	0.504861	0.696729	2.073955	1.950125	1.762214
H2AC19	1.032868	1.28E-37	59.96858	62.69181	64.37678	130.7234	121.3562	112.7681
SYNGR2	1.027254	2.71E-25	14.74149	13.36374	15.52236	29.40244	29.87997	25.34148
HSPB1	1.227406	0	1990.463	1935.271	1898.323	4483.848	4322.118	4286.141
SULT1A3	1.358442	0.000132	1.530857	1.330155	1.437796	3.312352	2.145178	3.893641
SERPINA12	1.899176	0.005151	0.058844	0.089394	0.719656	1.449428	1.202604	0.718414
AC112497.2	1.034875	0.000351	3.152972	4.647116	4.267519	7.629091	5.983707	10.05767
FAM171A2	1.168255	2.58E-05	1.59062	1.243368	1.284012	2.185031	4.412941	2.153386
KCNK6	1.633369	4.72E-188	10.52757	9.924629	11.10646	31.07072	31.77964	29.62394
AC005943.1	2.585251	0.005483	0	0.1605	0.487107	2.792518	0.431387	0.544796
KLF2	1.407839	2.12E-37	6.850315	6.086812	5.734297	14.12384	17.64855	15.62626
NXPH3	1.648398	4.14E-28	1.094546	1.301061	1.293205	3.630367	4.380707	3.294911
CLCF1	1.477967	1.33E-103	21.46816	20.3045	20.54597	54.88812	51.80735	57.82806
C8orf34	1.200197	9.53E-06	0.81651	0.893507	0.904251	1.970754	1.924894	1.50855
DLX5	1.043229	3.68E-08	3.792331	4.303939	4.984342	9.111587	6.894028	9.589946
AC092647.1	1.661483	0.001251	1.897759	2.301113	1.134442	5.865459	7.007378	3.623117
AL133346.1	2.453731	0.000218	1.187121	0.225889	0.256082	2.429034	4.203256	2.326496
VGF	1.711443	4.07E-09	0.860055	0.771482	0.586702	2.924552	2.286767	1.706527
LY6K	1.062542	8.08E-38	19.55416	14.39958	13.44154	33.85929	33.15781	39.34758
AC073111.2	3.488292	0.00434	0.119037	0.160442	0.045897	2.771422	1.038149	0
AC037198.1	1.260894	5.91E-34	20.39974	22.21451	20.38348	44.90337	50.60585	48.57458
AQP3	1.245932	0.001041	0.511685	0.84565	0.678757	1.445388	1.024179	1.535072
KBTBD11-O'	11.83464	0.002452	0	0	0	4.195159	4.066405	0
NPTX2	1.079331	2.34E-05	1.33626	1.258084	1.277965	2.378015	3.589607	1.965366
FSTL3	1.002315	3.05E-112	80.84367	80.9365	76.1557	154.9646	157.0962	142.1033
AC135050.2	4.56618	2.32E-08	0.140797	1.028132	0.34759	5.787788	11.93299	12.67529
DOK1	1.183473	3.77E-214	88.73403	87.10437	85.73126	184.5961	189.6856	177.7899
HAPLN1	1.556447	1.59E-17	1.206942	1.056972	0.943198	2.931469	2.386544	2.953698
FOXD1	1.203514	1.99E-40	20.15332	17.81336	16.84037	39.14339	41.6991	35.02168
KRT34	1.416882	1.72E-24	6.585554	4.822258	5.005813	13.94312	14.19083	13.63809
PRR7	1.015537	5.89E-06	26.17053	32.2075	22.06269	49.58036	63.84998	44.42338
SCX	1.292808	0.006926	0.588978	1.196392	0.725157	1.114013	2.976529	1.822114
RPP21	1.39833	0.006407	0.258828	3.829593	3.16272	5.76869	6.245282	7.615935
AC092807.3	1.204276	4.25E-10	2.993932	3.062183	3.773675	5.782846	5.857682	6.821973
MRAP2	1.515407	1.09E-41	7.325129	6.446267	7.362353	17.73837	18.12288	21.7314
PRPF31	1.757979	7.78E-05	2.782103	5.465057	2.77813	16.55444	8.46174	15.44547
IER3	1.051545	8.72E-08	4.917051	4.119914	5.937706	11.26669	11.5453	7.263818
AC103691.1	2.342796	0.011922	0.263571	0.250208	0.229667	0.436529	3.195634	0.452155
AC107223.1	2.063691	0.001979	0.212775	0.296118	0.208406	1.666731	0.950134	1.694325
SOCS1	1.17877	0.000196	1.221905	1.038241	0.697161	2.438439	2.897731	2.736366
ODF3L2	1.541334	0.004008	0.164218	0.441595	0.793188	1.213961	1.586429	1.211332
RAMP1	1.367438	2.24E-56	33.0948	30.06561	35.78818	85.75587	79.77818	78.48806
SLC43A2	1.436192	1.46E-34	1.63594	1.384557	1.805592	4.50868	4.223733	3.935894
COL4A2	1.029803	5.96E-198	34.014	34.06649	33.06482	66.84331	65.53049	63.78275
PAOX	1.128594	8.34E-05	1.218933	2.121776	1.382921	2.623577	3.151198	3.974769
AC110611.2	1.161229	0.00345	0.81766	1.215585	0.740332	1.869208	2.180941	1.944241
TUBB2BP1	1.012514	8.47E-08	5.442736	5.001083	5.622151	10.14118	11.27498	9.5386
PSPN	1.030377	0.000373	0.601427	0.462422	0.51485	0.892569	0.971179	1.231967
TGFBR3L	1.157685	1.99E-05	1.138414	1.651293	1.983275	2.63082	3.842504	3.124812
DACT1	1.050596	7.44E-05	0.867005	0.907255	0.648511	1.503997	1.297348	2.168533

ADAM19	1.107699	3.88E-11	0.895203	1.040373	1.115862	2.339221	1.965465	2.159399
TBC1D2	1.308655	2.22E-205	32.20029	32.84547	30.72836	76.06097	75.56402	73.18418
MFAP5	1.537782	1.26E-95	11.87686	10.639	10.44937	30.12437	29.87596	29.56119
CES1	1.352804	2.09E-20	3.353262	3.632268	3.662367	9.283174	8.030728	8.516479
KCTD4	1.255515	6.79E-09	1.579752	1.75676	1.678843	4.076663	3.448063	3.9205
CLIC3	2.304713	3.31E-17	1.620249	2.210562	1.81909	10.41514	9.75909	6.78519
C1QTNF5	1.443929	5.46E-07	3.458294	3.775984	1.432802	6.807823	8.991665	6.384237
TUBB2A	1.071816	2.98E-162	165.23	154.2448	168.4705	405.0309	332.7646	354.0017
MAP6D1	1.322133	3.60E-08	2.769679	3.915587	2.551882	4.547853	7.642766	7.033319
CRLF1	1.145574	5.13E-20	6.714061	7.078652	5.821312	13.24502	13.45268	13.29797
PLCE1-AS1	1.667984	0.000654	0.280254	0.257077	0.532648	1.495962	0.791218	0.976714
SERINC2	1.36702	8.32E-215	50.67709	52.54016	48.50258	129.3709	123.3655	126.5472
ENDOG	1.106496	1.64E-08	11.02124	8.745248	10.53316	15.53282	28.99183	17.67371
AL391056.1	1.707046	2.40E-09	1.202609	1.1109	0.594794	3.08231	2.540785	2.970417
CCK	2.746498	1.32E-06	0.143696	0.122413	0.523028	1.759282	1.809554	2.300413
SERPINB2	1.542641	9.86E-29	4.027488	4.002106	5.076991	11.68189	12.43039	11.92832
PLXDC2	1.076416	2.03E-10	0.538002	0.590849	0.536001	0.985366	1.155021	1.189896
AL031777.2	1.9376	1.82E-16	1.996434	2.04145	1.863728	9.075421	5.502488	7.261677
AL390726.5	2.412452	1.23E-05	0.4169	0.2322	0.153978	0.953743	2.194647	1.528253
LINC00547	2.64195	2.66E-20	0.302185	0.557069	0.337309	2.309054	2.00245	2.887151
TPM1	1.319027	0	530.5151	521.2827	543.5369	1272.174	1206.898	1347.916
SH2D5	1.163138	1.78E-06	1.413765	0.739934	0.987094	2.489863	1.864009	1.79499
EVA1B	1.270853	9.12E-35	68.77393	62.92777	60.35249	121.5232	172.5677	167.2692
KIF17	1.343089	5.05E-05	0.37554	0.293345	0.364929	1.644031	1.356792	1.116932
CKM	1.148922	0.008927	0.513387	0.623592	0.751421	1.995518	1.0824	0.967731
VSTM2L	1.422656	4.16E-39	10.02136	8.478556	7.245679	22.72409	22.71587	19.78218
NPB	2.191574	0.001156	0.072731	0.494272	0.200428	1.501702	0.937684	0.599866
AC004585.1	1.858124	0.002181	0.46337	0.331002	0.238563	0.794915	0.985254	1.891734
HACD1	1.16375	2.33E-52	17.70996	17.47962	16.59638	34.86167	38.3329	37.6072
RASIP1	1.075101	6.44E-06	0.948866	1.279268	1.574557	2.191929	2.522816	2.187327
C11orf96	2.193378	1.10E-21	1.735825	2.195378	2.672721	7.979097	11.93646	9.078921
ANTKMT	1.235713	1.60E-17	15.11962	16.72747	11.1592	28.66529	35.75673	30.18006
ZNF784	1.057074	1.14E-06	2.679335	2.301365	1.758919	3.820095	4.634781	5.027099
COMP	2.183375	0	17.72672	17.77714	17.76654	79.04727	74.51059	76.83221
CDH2	1.230385	4.38E-78	9.606912	9.982989	9.943829	21.44623	21.39613	22.97018
TSPAN33	1.030891	0.000196	1.217747	1.079136	0.592148	1.996931	1.611622	1.825688
IMPA2	1.787943	2.87E-51	7.502643	7.431742	7.609184	25.1225	21.30158	23.53421
PDXP	2.029484	0.005397	2.294969	2.262489	1.729087	4.074415	16.85307	3.520746
ZNF579	1.104593	3.88E-25	11.46361	11.58284	10.88369	20.62042	24.98199	21.20331
PXDC1	1.199948	6.10E-124	56.58008	58.9193	52.05097	122.1809	122.9228	121.0503
WAKMAR2	1.521533	0.001312	0.368862	1.127925	0.982587	1.34094	1.680641	1.582513
CLTB	1.143847	5.90E-103	166.787	178.3755	171.3059	338.0439	399.8748	343.7302
AC063952.1	1.127339	0.006523	1.248986	0.758069	1.618972	3.32299	2.223322	2.140905
LINC00862	1.249815	0.007095	0.404296	0.455828	0.751886	1.659326	1.198388	0.823041
AC002467.1	1.241078	0.000355	1.584491	1.820597	1.395662	4.42905	4.88421	3.243938
TNFRSF10D	1.208267	4.95E-184	37.96805	39.115	39.70181	87.26169	86.91532	83.04927
H2AC18	1.016748	7.93E-40	62.70002	64.17891	66.63289	131.8195	124.5814	116.8886
C5orf46	1.059659	0.011142	1.192994	1.824593	2.875775	4.291502	3.975455	3.731165
CD300C	1.969983	0.001924	0.233969	0.531571	0.071213	1.512047	0.716177	0.960399
Z95118.2	1.127938	4.12E-17	4.146798	4.281517	5.002552	9.621675	9.181211	9.18355
HSPB7	1.47962	5.80E-229	37.92314	36.84523	37.19946	99.59554	100.1246	100.2555
CAMK2N2	1.749515	5.47E-06	0.820236	0.594173	0.395106	1.912032	2.104326	1.819962
TM4SF20	1.589478	0.000887	0.207725	0.238727	0.553581	0.938396	1.387722	0.950918
KRT7	2.413652	1.11E-37	2.701377	2.908011	3.837985	14.7889	13.80574	17.12081
GNA14	1.24456	6.60E-13	3.262429	2.98478	4.227668	6.734784	9.281904	7.478467
FO393400.1	1.446154	3.62E-05	2.128387	4.057213	2.108108	6.441484	8.282668	6.856724
AL133476.1	1.970029	6.63E-05	0.530143	1.109	0.972881	2.480631	4.941724	2.392099
ULBP2	1.580182	1.45E-26	9.652596	7.792353	5.986129	21.59939	21.93493	23.24439

KRTAP1-1	1.194288	1.00E-13	6.493386	6.142887	6.185517	14.15954	13.96538	12.96814
PRPS1	1.278231	5.12E-187	45.56251	46.70829	44.25671	104.4705	107.8566	105.2673
CCDC200	1.341698	0.006283	1.331881	0.477594	0.904546	1.932688	3.258495	1.928852
ATP13A2	1.012795	2.01E-120	25.43207	26.34401	25.6005	48.88936	49.15623	50.26405
HSD17B14	1.541106	1.76E-05	0.866673	1.533963	1.116733	2.536581	4.095524	3.059258
PSG5	3.076292	1.51E-07	0.593081	0.069046	0.047223	1.645364	2.326407	2.393288
CARD9	1.203441	1.63E-07	1.855874	2.354714	1.363298	3.396775	5.611608	3.446771
LINC01013	3.097443	0.014674	0.528336	0.186068	0	2.654683	2.027127	1.382606
LINC00933	1.243986	0.001615	0.884333	0.445374	0.320056	1.363912	1.080997	1.434876
AC138894.1	2.793022	7.99E-07	0.021421	0.33286	0.141585	1.2622	0.709549	1.198165
EFHD1	1.645856	1.95E-83	17.56345	17.34086	13.76356	46.86053	48.68015	45.05068
TPST2	1.069322	9.41E-132	18.85257	20.22257	18.77605	38.09754	36.55817	35.32346
CDH6	2.197314	5.05E-21	0.370741	0.346527	0.385497	1.863901	1.320053	1.238295
TPM1-AS	1.695395	9.90E-05	0.163714	0.609785	0.255283	1.353847	0.953542	1.201025
IRS2	1.174555	1.99E-20	2.302526	1.857365	2.701675	5.111957	4.524802	5.129469
CIB2	1.024399	6.65E-05	2.079827	1.893452	2.051368	3.146039	4.126226	3.593947
AL590133.2	1.111623	0.012314	1.305372	2.115417	2.093052	4.586111	5.067349	1.852215
TM6SF2	1.674146	1.02E-05	0.635662	0.506075	0.665989	1.56232	1.717663	2.572244
AC006504.7	1.302845	0.00467	1.359212	1.899256	0.68877	2.446324	3.555321	3.366793
ARC	1.137934	0.001075	1.032619	1.023722	0.525964	2.606425	1.632561	1.149513
GDPD5	1.088078	4.27E-14	2.414849	2.335563	2.77317	4.945266	4.959023	5.000495
AC074290.1	2.751753	0.003928	0	0.34208	0.180426	0.707547	0.395176	2.455976
AP1M2	1.730647	5.79E-05	0.534149	0.488071	0.41245	1.819997	2.277709	1.427966
FAM174B	1.564661	1.75E-07	1.041606	0.677743	0.929592	2.107272	2.333164	1.605636
AC004967.1	1.359957	0.001637	2.706936	2.307355	10.22451	11.13555	14.34146	11.9597
PODN	2.0704	2.73E-21	0.706086	0.724469	0.668402	2.612945	3.425207	3.110876
TLL2	1.29015	1.04E-09	0.704913	0.467638	0.736188	1.529832	1.448674	1.72778
WNK4	1.580172	1.28E-13	0.936021	0.715546	0.611586	2.516647	2.010859	2.250939
HRAS	1.15041	0.000234	49.34702	43.71313	67.1712	96.13091	150.6765	93.26323
LIMS2	2.225506	1.85E-70	4.044944	4.463924	3.514519	18.0691	19.2406	16.54972
TRIM36	1.925578	5.42E-10	0.311966	0.546291	0.467025	1.915592	1.26799	1.633156
BMPER	1.271753	1.58E-108	9.39331	9.285568	9.851403	27.60382	26.42909	22.50793
LBH	1.100851	2.33E-42	9.79561	9.659297	9.134277	18.31701	19.79271	20.14161
DNLZ	1.428317	0.000103	3.363014	3.709367	3.124991	6.108411	13.30471	6.794725
NCF2	1.733735	5.21E-05	0.416403	0.181179	0.577416	1.507057	2.000077	1.785268
HSPB8	1.029564	3.42E-19	8.198202	8.690028	9.646729	17.86253	18.24082	15.54753
ESM1	1.118119	1.95E-07	2.541921	2.327167	2.800365	4.087569	4.674007	6.591395
C11orf52	2.122627	0.00021	0.518001	0.509731	0.925844	3.35351	3.05855	0.838864
SPHK1	1.757471	4.98E-264	38.44232	38.44574	38.90365	122.6523	129.7975	119.0402
CD36	1.087766	9.13E-29	7.391709	6.852867	5.912307	11.8435	10.91638	13.24736
NXN	1.137116	7.43E-60	11.97746	11.5497	12.43926	24.51498	24.54266	26.01997
ATP1B1	1.52246	1.96E-76	11.9528	10.15341	10.69282	29.60511	29.44373	30.91077
PGF	1.102365	2.57E-76	37.99908	38.80039	41.73856	84.25362	79.98837	79.31052
INO80B-WBI	8.752233	0.005962	0	0.000001	0	0	6.048986	0.785829
CST6	1.217577	1.83E-09	6.404011	5.739285	7.327637	14.79654	13.3506	15.09942
HIC1	1.040787	6.82E-42	6.114241	5.909037	5.181953	9.956372	10.49965	11.36219
SERTAD1	1.043723	1.14E-41	19.19667	17.97684	18.50675	39.75793	35.27325	34.3627
COBLL1	1.233217	8.52E-09	0.412117	0.819833	0.420556	1.345318	1.310745	1.340619
DBNDD2	1.148846	2.39E-08	4.524866	3.089335	4.259951	8.966187	7.270708	9.051363
EPB41L4B	1.449985	2.48E-12	0.755123	0.913565	0.781859	2.256548	1.92548	2.201036
RASSF7	1.53134	0.000306	1.157366	0.27911	1.164795	2.630045	1.915689	1.278109
HOXD-AS2	1.360881	2.94E-05	2.258159	2.146218	1.585687	3.124153	5.99256	5.651125
CNN1	1.278135	7.08E-17	5.427047	4.989326	7.16431	13.34028	15.28245	12.73536
GPRC5C	1.096937	0.001335	0.892642	0.565955	0.878007	1.488811	1.123498	1.650228
BATF3	1.215285	0.002349	1.702667	1.045683	1.296227	2.369148	5.23449	2.049504
UCP2	2.060604	0.00051	0.119879	0.379933	0.194379	1.026095	0.640162	1.494506
AC138028.6	1.243445	0.003697	0.698179	0.931948	0.508951	1.125567	1.409315	2.315785
NDUFAF8	1.018483	3.94E-09	126.5199	143.5439	107.136	214.6511	283.8888	231.9027

AC093536.1	1.963215	2.68E-06	1.906627	1.667096	0.770538	4.109729	6.792505	5.358141
LRRC73	1.211886	6.36E-05	0.741873	0.900743	0.662462	1.593542	1.768759	1.753132
FGF14	1.388924	0.005453	2.573161	2.169173	2.337025	10.15208	4.158363	3.346621
NT5DC3	1.250363	2.24E-65	4.026181	4.166293	4.342278	9.146077	9.5158	9.767095
PPP1R14A	2.926264	5.50E-72	6.471835	4.274156	5.035012	37.74732	35.37854	37.49141
KRT10	1.367808	4.12E-21	8.650121	9.65315	8.378706	17.71487	28.33015	19.64415
FBXL16	1.650253	1.39E-07	0.675278	0.368672	0.690376	2.074027	1.993084	1.516284
ATP1A3	1.619832	9.77E-09	0.396131	0.462527	0.558352	1.223693	1.359115	1.798272
PTH1R	1.107695	2.59E-06	1.309571	2.036818	2.343361	4.144051	3.691625	4.177347
OR7E38P	2.191147	2.77E-10	4.031658	1.781264	1.126758	7.760647	13.22303	9.20281
GPAT2	1.031556	2.19E-09	2.104513	1.808608	2.225246	4.256862	3.8396	4.121015
PMEPA1	1.221324	8.26E-20	2.691157	2.697176	2.009402	5.42409	5.875319	5.400334
KRT14	2.695903	2.21E-15	0.292969	0.506152	0.553419	2.606556	2.817769	3.203719
KRTAP2-2	1.477646	6.56E-05	1.118392	1.44129	1.219723	3.263693	3.359198	3.496396
AL357518.1	3.543522	0.002711	0.360571	0	0	1.428093	0.655511	1.989553
BAMBI	1.128398	4.99E-11	4.584962	4.570424	5.133794	8.106168	12.19932	9.602843
HAPLN3	1.051073	4.74E-23	7.406384	8.455555	8.251668	14.7025	15.79045	17.45327
AC016831.1	1.308797	5.20E-10	1.210389	1.281026	1.9787	3.792145	2.62183	3.696977
KRTAP2-4	1.635211	1.29E-08	2.052797	1.681295	2.074237	5.362524	5.711615	6.205196
COL4A1	1.043303	1.46E-121	17.10272	16.76985	17.10729	31.85257	34.00306	34.17321
NPAS1	1.810848	1.94E-121	18.84903	16.67966	17.44018	56.7114	62.34863	54.8792
SNX22	5.189591	3.60E-05	0.070069	0.16101	0.152777	0.160397	6.990337	6.694936
KISS1	3.775672	3.19E-16	0.296319	0.308674	0.450935	3.989796	6.941709	3.810789
SCARF2	1.389382	2.18E-96	16.75125	17.46211	15.19875	40.55024	42.91944	38.75407
C8G	1.540181	0.01689	0.473422	0.54552	0.428712	0.717883	2.009448	1.777591
CADM3	1.061877	1.46E-08	2.282728	1.584778	1.496872	3.709321	3.757636	3.206546
HBEGF	1.185529	2.32E-34	10.40565	10.53519	9.040658	22.38608	19.53377	22.98924
CSRNP1	1.177409	9.01E-21	4.012349	3.334487	3.572317	8.469336	7.597039	7.436128
FAM180A	1.043423	1.22E-21	16.53257	18.11535	13.90439	26.46543	33.04265	29.95924
CCL26	1.445445	0.000141	1.818406	2.559698	1.439541	4.412103	5.189957	5.217773
IL18	1.155593	0.001544	1.468923	1.675588	0.86581	3.885304	2.954684	2.369927
ANGPTL4	1.06016	0	406.5255	396.17	394.5275	811.2063	789.7592	778.6105
MUC12-AS1	1.262574	0.00338	1.584156	2.526067	3.421908	5.82233	6.910429	4.62666
NGF	1.559774	1.42E-10	1.760652	2.372138	2.232457	6.504351	7.694665	5.34643
MGST2	1.035382	1.91E-07	5.329476	5.351008	5.927304	9.64424	10.11117	10.36007
ADCY4	1.351313	5.03E-11	2.338235	1.193551	2.278616	4.772899	3.749532	3.767661
AC092042.3	1.951415	0.012834	0	0.862469	0.776156	1.124685	1.984836	3.085022
IQCJ-SCHIP1	1.547089	4.08E-37	6.227741	6.094089	5.858514	12.55079	14.04685	12.49672
AC124852.1	1.244548	5.75E-05	1.074817	0.7113	0.919984	2.075452	2.490069	2.304774
RGCC	1.192512	2.95E-08	8.586002	7.904281	10.97102	14.97345	27.84872	16.6041
OSCAR	1.208204	0.000968	0.468989	0.602618	0.816216	1.520724	2.481565	2.089306
PDE2A-AS2	2.434404	0.002089	0.279724	0.353588	0.095888	1.620691	0.488964	1.96946
SERPINA9	1.614275	2.35E-06	0.51053	1.129993	1.140151	2.639543	3.196768	2.151688
MEGF6	1.790783	8.24E-124	4.697698	4.259709	4.078003	14.76226	15.30696	14.25421
TSPAN13	1.361254	3.43E-40	10.25678	9.068324	9.781068	21.46584	23.71403	26.10792
C4orf48	2.179754	3.89E-11	41.77633	36.69719	20.53826	138.1807	179.4891	115.9295
NEK7	1.216579	1.33E-149	40.41145	39.81798	38.5193	81.14948	89.48062	92.40389
FAM110A	1.16319	1.66E-10	4.265939	3.375314	3.005771	7.251968	8.697483	6.709392
DUSP2	1.820574	1.99E-05	0.295655	0.461036	0.456114	1.613192	1.101089	1.597797
AC024592.3	1.18717	0.00022	6.849757	5.204228	2.658888	8.816747	6.13554	11.44561
CCN2	1.207522	0	334.4407	318.3728	313.4325	675.7723	737.4382	713.3289
PRELP	1.759252	3.60E-17	0.447445	0.486382	0.486111	1.605718	1.606289	1.38091
ACTA2	1.25382	0	576.7881	609.306	594.3441	1315.456	1303.152	1334.949
ADRA2C	2.264085	1.38E-20	0.904541	1.51519	0.825362	5.177039	4.428809	5.384899
SPINT2	2.045181	4.79E-09	0.543391	0.866149	0.813635	3.092007	4.038128	1.845094
AC013652.1	1.013346	0.000494	1.234189	1.86421	1.956753	2.739667	3.680538	2.831045
BCL7A	1.093115	3.51E-10	1.387693	1.146229	1.309635	2.468159	2.362782	2.860354
FST	1.820817	0	71.95318	70.76162	67.16303	227.7318	232.2775	241.0368

FOXC2-AS1	1.066146	0.000163	5.761863	7.432398	5.774474	12.43858	11.89229	13.66332
KRTAP2-1	1.546849	2.57E-05	1.129037	1.312517	1.126214	3.81424	2.929634	3.388716
HYI	1.950904	2.79E-11	1.346671	1.403964	1.268779	4.813273	6.922811	3.459464
HS3ST2	1.717892	6.70E-11	0.663382	0.970388	0.712494	2.502651	2.535333	2.371685
NRG1	1.136954	2.61E-16	7.640741	4.607584	4.714736	14.99128	20.94198	12.32018
ELN	2.347304	3.71E-73	2.286319	1.74653	2.395484	9.336047	10.26007	8.678118
RBM24	1.084774	3.82E-07	1.950537	1.257001	1.622589	3.307358	3.230677	3.104964
AL135925.1	1.040071	0.00033	1.268647	1.145997	1.099616	1.868479	2.454096	2.580519
HES1	1.551537	1.81E-07	0.914811	1.11289	1.124965	3.279637	2.89466	2.542288
EGR2	1.459493	2.26E-09	1.156891	0.805176	0.743974	2.434194	2.341848	2.090304
MSX1	1.426275	1.04E-27	5.493173	4.95655	5.026613	11.92496	15.09391	12.9517
AL137186.2	1.247023	0.002262	0.206091	0.800366	0.897485	2.94702	1.310979	1.947571
PKMYT1	1.419601	4.88E-17	3.647853	2.937513	3.101865	7.163073	9.4277	10.18252
CDT1	1.178952	2.31E-11	2.641492	2.561448	2.326849	5.628344	5.740493	4.19426
ANKRD1	1.779243	5.93E-244	38.00285	35.1377	39.33646	118.4275	126.4263	123.1197
AC107214.1	1.071245	0.009209	1.436817	0.764234	0.852655	1.943797	3.634994	1.62749
AUNIP	1.092196	0.012569	0.248007	0.784551	0.921562	1.090488	1.422436	1.507084
TAFA5	2.254081	4.18E-09	0.431295	0.323399	0.20817	2.037266	1.375134	1.064598
KRT16	3.756981	1.45E-08	0	0.369158	0.134213	0.982164	1.412923	1.694211
ABHD17AP6	1.436462	0.007482	0.6894	0.225298	0.625055	1.545058	1.31667	1.219551
NUDT8	1.038057	0.002611	1.749755	2.615036	3.773138	3.87796	5.076799	7.729578
AC006538.2	1.131037	0.002273	1.872092	2.175711	3.218198	6.16456	5.066314	4.08417
GABBR2	2.483885	4.12E-41	0.410449	0.551897	0.588562	2.858079	2.753746	2.63796
TUBB2B	1.10383	1.87E-114	52.71972	52.45236	49.1414	58.44634	94.78807	72.13512
AC034223.2	1.053463	0.013044	3.826694	2.434487	1.819019	5.867615	3.604953	6.479038
LINC01711	1.247476	0.000306	1.675838	1.020933	1.727998	2.757707	3.564101	3.712777
CCDC81	1.005961	2.34E-07	3.374447	4.189334	4.444061	8.631032	6.832956	5.85252
SAC3D1	1.336091	2.64E-17	5.038879	4.759313	4.611343	10.48785	13.63261	11.26805
P2RX5	1.426539	1.97E-15	2.879614	4.820663	3.57809	10.06929	10.13434	9.228436
ATP10A	1.0379	3.42E-29	4.74743	4.51472	4.125097	8.386897	8.205144	9.167174
DLX2	1.164846	0.000926	0.573499	0.643766	0.405169	1.077581	1.224638	1.030003
AP001107.3	1.083943	0.004709	1.769467	1.794887	3.247869	4.940583	4.309482	4.651122
GAL	2.244593	0.0041	0	0.466641	0.698406	1.043347	2.332345	2.026868
SLPI	1.250884	0.009819	1.210552	2.832723	1.201217	2.025978	4.887194	5.101274
AC026356.1	1.03181	0.011227	0.794709	0.834337	0.528112	1.322997	1.379526	1.533812
TSPAN18	1.155592	5.58E-10	2.985542	2.149986	2.810718	4.440852	5.292121	7.022195
PLAAT1	1.505134	0.000364	0.764188	0.795986	0.745323	1.499521	2.250015	2.794392
OXTR	1.974488	1.76E-24	0.80727	0.925646	1.221561	3.721136	3.722224	3.188697
IL7R	1.094287	5.37E-31	5.120779	5.614883	5.700808	9.851834	11.47744	11.34267
AC109479.1	1.058404	0.015905	2.867013	1.34896	1.143837	4.041743	3.92015	2.72609
CRYAB	1.561367	5.41E-303	124.879	155.5831	116.4794	420.2161	430.5326	454.9485
KIAA0040	2.312262	7.54E-18	0.390409	0.332674	0.245629	1.304323	1.740888	1.580645
AC110792.4	1.935039	9.71E-10	0.304368	0.465041	0.486301	1.596846	1.355672	1.648915
INAFM1	1.165965	3.28E-31	21.48549	19.14149	18.93144	42.00301	44.17211	41.19049
GAD1	1.197385	2.22E-05	0.598103	0.60948	0.464849	1.393759	1.465721	1.159533
POPDC2	1.06921	0.001699	0.735842	1.209958	0.879244	1.632699	1.770705	2.302696
CTU1	1.49792	1.07E-15	3.590226	2.702882	2.200685	7.18711	8.614555	7.075646
KRT18	1.046355	6.42E-10	4.997036	4.334398	4.004702	9.375601	7.776269	8.893106
MMP23B	1.03422	0.007391	1.450582	1.556648	1.149878	2.203815	3.988339	2.526931
IGFBP3	1.878882	0	369.4818	358.0906	359.9958	1236.596	1259.368	1227.664
BMP6	1.677762	1.95E-110	6.165086	6.040525	6.224366	19.4715	18.16677	18.57767
KRTAP2-3	1.604529	1.86E-63	18.56749	17.76099	15.93726	49.62223	51.27161	50.58239
CITED4	1.446993	5.95E-19	12.89991	12.01216	8.069204	23.74133	34.4149	27.50879
TMEM51	1.138732	2.28E-08	2.03647	1.898856	2.135281	3.803221	4.379853	4.29565
PDF	1.023197	1.04E-11	4.158204	4.334245	3.311715	6.788857	8.847119	7.220557
WFDC1	1.197506	2.20E-20	7.936054	7.65636	6.539908	15.92946	18.6721	17.3318
DGKI	1.16994	6.42E-16	0.683379	1.365713	1.315067	2.218152	1.700646	2.514335
AL031058.1	1.367123	1.07E-07	2.473036	2.133129	2.553511	5.261955	6.227465	6.156254

LYPD6B	1.527947	6.45E-08	1.605088	2.036881	1.206124	3.997545	5.12625	3.58186
SPDL1	1.025389	1.74E-46	16.94966	18.71701	17.30891	34.59425	31.80235	34.98499
HSPB2	1.14584	4.88E-31	21.51208	20.37003	21.27684	47.13004	41.16266	44.94597
AC099066.2	1.157577	1.68E-10	5.172915	5.564105	6.331421	11.89838	14.15389	9.943305
RAD51	1.220454	1.13E-08	1.697171	2.078774	1.611706	4.498435	3.375902	4.096942
NELFE	1.235134	2.52E-22	9.331059	14.16326	13.63107	34.05222	26.00305	34.74596
EDN1	2.403241	4.26E-53	3.2476	2.78264	2.061238	12.64018	13.36197	14.81626
NAP1L2	1.110025	3.94E-07	1.609294	1.309072	1.387246	2.58261	3.428377	2.851093
CTXN1	1.053018	8.71E-08	6.082662	6.74013	6.142626	9.705133	16.64648	11.22346
CHCHD10	2.13981	3.00E-09	13.45773	11.9476	12.6857	38.63719	79.58337	39.31307
CALHM6	1.731056	0.000363	0.417835	0.477513	0.487507	1.992119	1.043199	1.511636
TAMALIN	1.628471	0.00059	0.439512	0.429419	0.226849	1.639235	1.127817	1.25411
IGFBP1	1.064031	1.55E-13	6.239703	7.352821	5.697206	12.09259	12.3346	14.04934
VLDLR-AS1	1.29726	0.000826	0.408774	0.32606	0.971438	1.707886	1.566089	1.554712
SHISA9	1.253018	1.71E-06	0.507323	0.520115	0.417027	1.206816	0.766137	1.174292
VCAM1	1.012026	3.19E-135	67.15425	62.47563	69.2712	125.6482	126.968	129.645
MMP3	1.153856	0.008324	0.345567	0.739252	1.474047	2.087788	1.947252	1.447367
NR4A2	-1.073172	0.00283	0.681922	1.220659	1.103959	0.453509	0.522174	0.345589
ALG1L6P	-1.301537	0.006945	2.502461	1.779796	1.667894	0.673532	0.832124	0.747118
LAMA5	-1.530257	2.19E-58	9.7032	10.14582	9.060545	3.598033	3.349337	2.917261
FMNL2	-1.304306	2.54E-34	7.116781	7.079219	7.133754	2.386988	2.993815	2.805864
CLIC4P3	-1.811632	0.000124	2.662445	2.896766	2.001231	0.906504	0.593069	0.53898
FAM20A	-2.26884	1.31E-41	4.352582	3.841893	3.844952	0.77635	0.719552	0.799926
CFAP69	-1.382197	0.000108	1.042905	1.832102	1.491017	0.256713	0.830997	0.697412
PAN2	-1.081872	2.36E-08	3.497202	3.66255	3.169441	2.022693	2.768536	1.926692
FADS2	-1.448252	2.79E-219	84.63409	85.34663	78.4927	30.4917	28.3353	27.45691
TNFSF15	-1.085707	6.64E-17	3.566546	3.448227	3.13652	1.396167	1.649395	1.468889
IGF2BP3	-1.125652	4.95E-13	5.943697	7.084783	6.756183	3.005958	2.472722	3.064785
PBX1	-1.16418	1.55E-06	1.518561	1.103727	1.825824	0.404161	0.961375	0.605281
CFAP126	-1.126728	0.004028	3.093175	2.581246	2.617438	1.279339	1.284888	1.026145
TRIM52	-2.1941	6.98E-33	4.768572	3.581579	4.412117	1.015131	0.857442	0.720016
COL6A3	-1.186109	0	1063.386	1053.795	1023.585	442.8373	426.8762	438.6505
PIGCP1	-1.086128	0.003201	3.914889	3.496751	3.093412	1.110494	2.292385	1.285578
ZNF608	-1.084643	4.52E-09	1.885365	2.710387	1.76087	0.808295	1.69109	1.788247
PDE4DIPP2	-1.387593	5.33E-50	9.447245	9.147229	8.79771	2.908798	2.82937	3.14155
AC055811.4	-1.667623	0.000347	1.949875	0.855187	1.640931	0.653612	0.260545	0.407471
RPL32P3	-1.595638	5.03E-11	2.818415	2.642325	2.72807	0.724167	1.15416	0.895624
RBBP4P1	-1.455117	0.00899	1.363921	0.951267	1.512535	0.564078	0.149272	0.58615
SEMA6D	-1.118724	3.87E-05	1.370776	0.848984	1.155415	0.389073	0.346153	0.59045
NFE2L3	-1.290128	1.04E-25	8.23913	6.781681	7.77868	2.711897	3.145475	2.919383
PLAAT4	-1.387101	1.66E-22	25.57892	23.02917	22.6363	8.841117	8.434226	8.41618
CRABP2	-1.059897	2.33E-44	75.7878	73.34434	68.71211	34.96223	32.86189	31.80684
WASH5P	-1.079068	0.00743	1.023276	1.308883	0.679841	0.479497	0.787303	0.487829
MTATP8P1	-3.605469	1.17E-07	8.785306	3.03817	23.70346	0.750421	0.807403	1.01776
LINC00342	-1.01676	2.97E-07	2.837718	2.945926	3.086527	1.498857	1.249587	2.177534
SLC1A3	-1.031746	3.51E-09	3.761571	3.464721	3.639341	1.633219	1.590925	1.687384
PDE7B	-1.088638	1.79E-29	7.816706	7.564956	6.996738	3.103087	3.376405	3.421515
SAXO2	-1.214812	0.009289	1.474418	1.267132	1.117173	0.308576	0.504639	0.254282
ATG16L2	-1.724001	6.80E-17	7.959708	5.549235	6.470154	1.917407	1.140882	2.084475
PAPPA2	-1.026012	9.70E-39	5.940723	5.92219	6.250195	2.73543	2.935506	2.801915
AC079949.1	-2.233589	0.000418	0.45747	5.204355	0.501098	0.885632	0.069501	0.103348
SV2A	-2.334634	5.78E-24	2.41527	2.441845	2.7181	0.604167	0.370604	0.422046
AF201337.1	-1.320726	0.005819	1.855085	1.855703	2.767859	0.689328	0.765258	1.025553
MT-ND4L	-1.022531	7.00E-190	5727.911	6061.287	5881.794	2758.311	2625.121	2909.645
LINC02057	-1.192786	0.012007	2.318654	2.634126	1.438543	0.895186	0.691933	0.863645
GRIA1	-1.332451	7.73E-11	2.483612	1.958363	2.992285	0.846132	1.096462	0.924659
AC013451.2	-1.750512	0.002101	1.514457	2.149559	1.572759	0.512405	0.467604	0.438723
OAS1	-1.289234	6.31E-10	4.646823	3.752046	4.206265	2.354012	1.72791	1.214904

APOBEC3D	-1.098667	1.10E-05	2.314112	2.211108	2.491726	0.837946	1.169035	0.944471
NLRC5	-1.015177	8.60E-22	16.34899	13.75336	13.8017	11.07006	8.041949	6.092352
ITPR1	-1.284587	2.05E-12	2.684232	3.371575	2.701759	1.490832	0.856388	2.204662
SAMHD1	-1.092451	2.35E-39	15.95655	15.14086	14.70025	7.199932	5.929917	7.166686
SMG1P1	-1.091565	0.006351	2.039352	1.086677	0.947901	0.324248	0.563717	0.4859
CCL13	-1.179589	1.13E-05	6.223515	7.357884	5.949936	3.238228	2.774379	2.171834
ERV3-1	-1.051964	8.59E-06	2.534968	3.987948	3.433985	1.492006	2.00138	1.782229
CD74	-1.022985	1.46E-109	153.7226	152.688	136.2721	67.52358	62.64562	65.50102
BDKRB2	-1.218523	2.27E-22	7.477731	7.241728	8.758337	3.841443	3.32974	1.838475
OGT	-1.212475	2.09E-129	28.77296	28.97567	27.31775	12.4714	11.70889	12.58616
RMRP	-1.548074	0.002317	7.594936	4.865911	21.42582	4.509152	2.94657	3.415761
RPPH1	-1.833	0.0164	2.80125	0.718036	9.30367	0.776664	0.83143	1.734851
ADAM33	-1.585271	2.24E-10	2.795721	2.707214	2.20831	0.897594	0.599473	0.648188
BORA	-1.007455	0.00258	0.962549	1.034375	1.562081	0.524744	0.640128	0.532988
HR	-1.653728	6.36E-15	1.939096	1.751776	1.884363	0.476628	0.768369	0.507285
C1RL	-1.509351	8.35E-46	12.21532	12.98965	12.52419	4.688118	4.246404	4.069692
SEMA3A	-1.466889	1.57E-21	3.084515	4.392798	3.963529	1.623924	0.989811	1.473137
RBM26-AS1	-1.165541	0.002545	1.60196	0.639795	1.243074	0.397378	0.691197	0.186894
TATDN2P2	-1.130042	0.012125	1.235469	0.900247	1.05148	0.650522	0.527934	0.204861
ITGA8	-1.139867	3.65E-05	1.16646	1.078161	1.001808	0.363151	0.763445	0.354667
ABCA1	-1.533623	2.31E-35	3.725533	4.055747	4.172588	1.110264	1.541478	1.275797
ZNF841	-1.196469	2.11E-15	7.100673	5.888816	5.462208	1.953673	3.011015	2.202355
ETV1	-1.155233	6.26E-20	6.812159	7.769726	7.835388	2.351538	3.629101	3.484771
GNG12-AS1	-1.284616	0.003059	2.803896	1.793414	1.440999	0.311199	0.683771	0.446189
PHC1	-1.222168	1.30E-18	4.350574	4.756704	5.385974	2.196825	1.730241	1.81252
COLEC12	-2.400429	1.03E-19	1.808609	1.24643	1.2416	0.261352	0.22518	0.276773
AL135999.1	-1.092452	1.76E-06	3.297558	4.451718	2.736935	1.811323	1.682465	1.181608
EREG	-1.167788	5.89E-16	4.739731	5.793908	5.254065	2.044145	2.541554	1.963109
HERC2P7	-1.468271	0.005707	2.712939	1.641028	1.610103	0.538219	1.414384	0.642936
PARP9	-1.032431	7.07E-18	12.96367	12.40065	15.31662	5.434261	5.13348	7.53553
C16orf86	-1.267409	0.000566	1.376897	2.740493	2.604407	1.133407	0.856413	0.761295
FADS1	-2.09288	0	57.06151	57.19734	57.87702	11.80359	13.2426	13.47973
TMCC1-AS1	-1.101539	7.36E-05	1.366485	1.331594	1.783762	0.633605	0.538768	0.94081
LDLR	-1.279413	3.04E-92	31.7699	33.51084	33.47034	11.1647	11.72955	13.00576
INTS6L	-1.194432	2.44E-05	1.289109	1.249421	1.820077	0.743317	0.515083	0.495183
LSP1	-1.374776	6.02E-18	8.890187	8.435939	7.310502	2.763781	3.337367	3.135726
ANGPTL2	-1.589819	1.57E-36	8.290825	7.870633	7.848144	2.445821	2.52564	2.515073
SLC35E2A	-1.408359	2.42E-06	3.263659	3.854266	2.488156	1.296899	1.101599	0.691662
AC006058.4	-1.619958	5.29E-18	8.783349	9.558028	8.304752	2.19794	3.400548	2.661556
PARP14	-1.09222	4.06E-34	6.274458	6.773336	6.415446	3.079599	2.711524	2.869742
FAM86C2P	-1.353862	0.0014	1.6738	2.118694	2.251205	0.296503	1.006909	1.137587
RGS17	-1.092872	2.50E-07	1.008586	1.123096	0.95355	0.531147	0.411416	0.430173
THSD4	-1.070799	4.26E-45	11.46414	12.52393	12.17739	4.905788	5.419389	6.018462
PKD1P4	-1.238464	1.25E-23	5.944785	5.921243	5.693528	3.246067	1.826568	2.178583
AL133243.2	-1.478512	0.001518	1.859651	1.236452	0.790027	0.337726	0.539768	0.431808
RN7SL4P	-1.55796	0.016755	2.883912	2.210583	4.739069	1.407407	0.621918	1.076041
IFITM1	-1.337664	4.08E-141	236.3807	222.8467	237.8749	91.55357	96.4072	88.96491
FP236383.4	-1.146588	0.000115	1159.313	1339.965	2131.467	742.1406	632.1463	622.249
AC004890.2	-1.321884	7.26E-07	4.181506	3.103171	4.528963	1.279165	1.70021	1.877348
LINC00106	-2.700151	0.000516	1.790474	2.094708	2.590493	0.352064	0.229106	0.244965
DPP4	-1.245233	7.90E-14	3.504494	2.752416	3.41571	0.761863	0.747392	0.793719
TBCE	-1.70979	0.000105	1.929955	2.163261	2.129358	0.213225	0.442845	1.10472
MAT2A	-1.050386	1.01E-174	150.3665	148.4252	148.1281	63.11224	66.1689	67.76247
CEP126	-1.001622	4.67E-08	1.575651	1.559197	1.381933	0.745759	0.767769	0.619506
PPL	-3.028038	1.94E-274	13.8545	15.03904	13.28245	1.622218	1.634918	1.734912
DMTF1	-1.046672	3.98E-21	13.06272	14.07339	12.60277	6.656766	5.750267	7.073354
RPARP-AS1	-1.497494	1.18E-06	3.819781	3.514606	2.087923	0.882472	0.695652	1.145383
AC026316.2	-5.965667	0.000696	1.457941	0.708301	1.099038	0	0	0

RN7SL1	-1.195063	0.010742	43.61008	51.11129	117.2985	33.38958	29.30911	25.61541
NEAT1	-1.376914	0	52.34847	56.87014	58.79575	24.23051	30.33218	12.67376
MAP2	-1.397308	8.84E-13	1.980456	2.036374	2.794941	0.571275	0.658429	0.719449
AL691432.1	-2.098057	3.35E-05	1.671872	1.66994	2.074017	0.367139	0.253642	0.544008
C3	-1.729164	3.62E-14	1.853157	1.812094	1.759064	0.393839	0.519075	0.654719
STEAP4	-3.646274	0	37.84013	38.49237	39.84678	2.886322	3.053854	2.945544
SAP30-DT	-1.273054	0.000125	4.017231	4.117408	3.198687	1.638102	0.915634	1.377126
TGFB3	-1.224673	3.27E-27	9.756076	8.730069	8.926619	3.40594	3.75778	3.844882
LINC00943	-1.898915	5.44E-08	1.807335	1.471231	2.236427	0.579917	0.570083	0.328109
SULT1B1	-1.196839	2.10E-17	3.571353	4.488547	4.417706	1.286555	1.38519	1.496036
SEC31B	-1.193513	1.43E-06	1.718457	1.939759	1.776557	0.575815	0.589169	1.117709
GABRE	-1.239623	1.58E-17	4.941033	4.680898	4.53166	1.861067	1.715173	1.627871
OLFML1	-1.690493	9.85E-52	19.83603	18.07286	15.5345	5.625872	4.879337	5.539491
HMCN1	-1.028457	2.07E-15	1.294385	1.255448	1.146703	0.515651	0.561384	0.517745
CCDC18-AS1	-1.985527	3.04E-13	3.359358	4.246449	3.016855	1.147031	1.426954	1.170088
TRIM9	-1.258026	4.69E-05	1.640121	1.344792	0.880976	0.359587	0.487195	0.620267
CHI3L2	-1.452219	2.20E-08	6.035747	5.548882	9.096463	2.935976	1.563909	2.816947
NTSR1	-1.252833	3.28E-14	5.265121	6.141325	5.447428	2.815233	2.14535	1.770768
ZNF788P	-1.613213	0.000932	1.857637	1.43812	2.240529	1.077431	0.461616	0.325101
APOL1	-1.074608	6.28E-97	58.09941	58.32953	57.18555	25.66982	27.5553	25.98169
H2BC19P	-1.170353	1.56E-05	1.197305	1.220881	1.474537	0.681608	0.584919	0.653506
CFI	-3.416276	0.017624	2.874787	3.161642	5.10865	0.378954	0.459691	0
IFIT3	-1.259286	5.79E-58	24.40124	23.12222	21.84427	8.494024	8.591345	9.801459
GALNT15	-1.43972	1.01E-27	5.896709	7.405518	6.47234	2.657989	2.430084	2.562403
EPSTI1	-1.004797	1.50E-25	16.68047	14.62732	16.91339	7.413612	7.714088	8.393173
RFX8	-1.08759	6.44E-13	9.423514	8.903318	8.521209	4.773535	3.533243	4.390797
RN7SK	-1.301375	0.000147	17.07145	13.76493	39.38783	9.922336	7.31727	9.922235
SCAND2P	-1.024338	8.32E-07	2.58783	2.56406	1.788717	0.750341	0.959992	1.149244
LINC00106	-2.536279	0.000411	1.941665	2.773862	3.143269	0.580359	0.229136	0.278258
CASP1	-1.078921	4.57E-05	3.435021	3.393154	2.236835	1.391181	1.187785	1.712077
AC092683.1	-1.145439	1.06E-09	5.98948	5.469696	5.218134	2.077044	2.442057	2.762231
ARHGAP26	-1.059876	7.47E-11	2.061116	1.807604	1.875497	1.026381	1.168232	0.762128
BTN3A3	-1.115311	1.08E-21	15.81196	17.36745	18.49328	7.948624	5.931811	8.096258
PSMB8	-1.099644	2.96E-07	13.14443	8.724192	11.3172	4.049519	5.953512	4.559698
CRYBG1	-1.032152	8.90E-90	19.53718	19.65618	19.37267	9.515079	8.449652	9.049143
ZNF354B	-1.045989	1.03E-05	4.035356	3.46566	3.473897	1.374029	2.240862	2.293654
CCL7	-1.918855	3.98E-05	3.79915	2.551782	3.978031	0.363462	0.922064	1.320546
ZNF436-AS1	-1.138794	0.003785	1.387123	2.021445	1.403026	0.976457	0.345817	0.958094
AL355032.1	-1.011006	3.00E-16	122.4699	116.8473	117.5219	69.09486	54.31848	45.18363
TMEM178A	-1.411086	0.000137	2.047795	1.584901	1.389867	0.644577	0.57636	0.572191
AC108010.1	-3.402503	3.33E-17	2.482324	2.943209	3.666037	0.418178	0.235065	0.146533
RNF213	-1.130736	5.95E-109	13.56651	9.683216	11.81023	4.671955	8.047079	8.348992
IGFBP5	-2.106644	0	427.3391	431.3367	420.2294	94.07947	95.97454	93.02414
MTSS1	-1.261945	4.96E-139	33.81285	32.14381	32.63297	13.25191	12.76301	13.27737
PDPK2P	-1.28809	2.94E-05	1.572856	1.769381	1.358321	0.683983	0.539815	0.5897
TMEM147-A	-1.251959	4.69E-07	2.725919	2.430684	2.109312	0.827077	1.341002	0.698039
NID1	-1.104918	6.28E-228	70.61306	69.07199	67.65096	30.44113	30.1814	31.39425
EIF2S3B	-1.054368	0.001052	3.12968	3.182968	2.082271	1.464855	1.525237	0.842207
CA9	-1.089501	3.06E-08	6.064091	5.112475	6.065847	2.556559	2.710818	2.428727
FLT3LG	-1.084597	2.87E-06	7.976079	10.14946	8.032842	5.01399	3.596602	2.942857
DIO2	-1.132721	6.97E-05	1.535541	0.982298	1.010565	1.019136	0.54209	0.363439
MALAT1	-1.195388	5.19E-19	30.90463	32.98475	40.20961	15.11448	15.54311	14.28307
OLFML2A	-1.075021	5.09E-50	13.70099	12.34624	11.39379	5.871446	5.645918	5.379311
ZNF559-ZNF	-1.572274	3.80E-05	1.283547	1.904875	1.273825	0.329308	0.597845	0.541182
IFIT2	-1.207652	1.25E-14	4.752791	4.411334	4.88166	1.90866	1.768542	2.185012
CD14	-1.0788	0.001128	3.189538	2.219634	2.546435	1.094702	1.655955	0.851226
SLC6A15	-1.432937	8.55E-12	3.275474	3.187882	2.917279	1.399983	1.170199	0.755922
KRT19	-1.667349	6.63E-92	47.69409	46.00752	44.06353	14.29035	13.65911	13.05247

PKIB	-1.394385	3.27E-08	5.842163	6.138466	6.760097	2.496646	2.891187	2.034337
FAXDC2	-1.393644	5.27E-12	3.656295	3.759387	4.204658	1.32956	1.010057	1.535625
XIST	-1.150961	1.89E-60	14.63637	10.8871	10.75105	7.285508	4.869539	5.766761
AL360181.3	-1.507838	0.001856	0.983352	0.653653	2.185025	0.534199	0.470915	0.308139
NFAT5	-1.152179	1.65E-14	2.801418	4.713687	4.07693	1.437846	1.841267	1.752941
ZSCAN12	-1.365631	2.79E-06	1.227593	1.4762	0.848726	0.27099	0.433496	0.381583
IFIH1	-1.432899	2.07E-07	1.601972	1.962785	2.449682	0.683737	0.563425	0.864999
GABPB1-AS	-1.07371	1.09E-07	2.2459	1.843254	2.155537	2.212607	1.59713	1.460501
SHPRH	-1.132336	9.06E-07	1.184863	1.184116	0.833038	0.488864	0.447532	0.354665
XAF1	-2.077548	0.012058	12.9351	11.60775	12.87999	5.36355	0.602504	2.231128
BTN3A1	-1.077291	5.04E-15	6.762236	8.740749	7.763164	3.503124	3.087486	3.921529
APOL3	-1.070196	2.53E-21	22.96004	24.07163	17.97083	9.213626	8.643774	9.78999
AL355607.2	-1.362328	7.72E-08	7.634314	7.948564	5.533465	2.259907	2.394832	3.155976
DYNC2H1	-1.061966	9.85E-27	3.529228	3.850636	3.365673	2.243675	1.660214	1.777139
AL096711.2	-1.066187	2.61E-09	4.315398	5.004983	2.240121	0.991731	1.116629	0.958303
PPFIA4	-1.159503	2.49E-29	10.76959	10.07835	10.37223	3.567884	3.5772	2.923712
AC007192.1	-4.067047	5.87E-19	30.62786	16.60832	10.99331	1.284552	1.153285	0.849578
PLA2G4A	-1.390554	1.17E-12	3.610109	4.385327	3.946826	1.267124	1.339046	1.729851
ALDH3A2	-1.061913	7.58E-88	35.83059	36.35764	35.17026	15.31204	16.97746	16.4163
AEBP1	-1.659432	8.89E-69	11.34847	11.48912	11.72587	3.432642	3.743541	3.619429
FP236383.5	-1.138585	0.000658	1161.056	1334.406	2254.877	791.0832	640.9121	627.7267
SOD2	-1.063125	1.98E-130	61.71835	80.24098	64.58168	37.20661	34.19416	34.6824
AC006486.1	-1.467713	0.013984	2.28213	2.526277	1.625987	1.343119	0.45133	0.373555
ADAMTSL4	-1.5729	4.12E-42	11.51516	11.32952	12.92082	3.636289	4.335692	3.26079
DNAH1	-1.064399	5.07E-08	1.692016	0.711204	1.061838	0.472225	0.69251	0.377285
PRKX	-1.006376	1.04E-09	3.987932	4.156514	4.971793	1.68713	2.089399	2.309479
ANKRD10-IT	-2.923213	9.60E-06	1.303545	1.394924	2.496151	0.290876	0.063796	0.250108
SEMA5A	-1.02175	1.22E-41	7.988256	7.392756	7.545502	3.460299	3.228137	3.804666
LDB2	-1.362399	0.000837	1.126815	1.795407	2.164986	0.366405	0.567684	0.939085
PCCA	-1.006828	7.23E-10	5.152466	4.854769	5.283628	2.697406	2.325398	2.479095
FER1L4	-1.285184	3.01E-45	17.6574	16.27901	14.69622	5.898157	7.410594	6.334859
ZNF731P	-1.268991	8.45E-07	8.899942	5.846657	9.641328	2.953713	2.163437	4.816086
SMARCA5-A	-2.539475	0.000355	0.82706	1.421711	1.100375	0.29784	0.153267	0.080924
GPC6	-1.133722	1.11E-14	3.342628	2.961044	3.4258	1.274076	1.272335	1.689919
HERC6	-1.202868	2.31E-18	6.235258	5.442507	5.92889	2.19445	2.281252	2.736081
IL18R1	-1.109226	0.000931	1.368802	0.852943	1.143301	0.283594	0.616416	0.488199
POU6F1	-1.092773	3.16E-06	1.815963	1.73421	1.327792	0.589508	0.734271	0.531901
CD302	-1.055512	2.25E-08	3.319956	3.734423	2.666974	1.314632	1.445366	1.702405
GRTP1	-1.06306	1.90E-06	4.316447	5.661703	4.483789	2.177083	2.188621	2.053988
BX255923.1	-1.477852	0.015221	3.645474	1.30793	1.418496	0.556952	1.082871	0.626422
C1R	-1.940062	5.06E-114	35.99147	30.51312	33.04945	7.427123	8.364137	8.150741
SPATA6	-1.054534	1.84E-06	1.763395	1.912409	1.579163	0.773093	0.599788	0.782854
COL21A1	-1.515732	3.37E-22	4.096635	3.916379	4.292655	1.268297	1.827709	1.295475
MOXD1	-1.934292	1.96E-119	28.90222	31.41847	32.65504	8.438357	7.283	7.442807
HLTF	-1.828602	7.56E-32	5.442931	7.347409	5.928931	2.247827	1.513408	2.105256
MME	-1.375129	2.25E-158	40.36329	38.67839	39.31284	14.70234	14.45602	14.55611
HERC2P9	-1.597986	7.20E-12	3.702748	3.084467	2.194118	1.096642	0.897993	0.83253
PAPLN	-1.082186	2.45E-09	2.071025	2.172226	2.331837	0.855523	1.144097	0.786189
S100A4	-1.085934	2.62E-30	140.4159	129.5173	153.4404	61.99741	70.91493	55.58875
AC018647.2	-1.171024	2.79E-08	1.905348	1.672176	1.756091	0.775122	0.726397	0.747552
KYNU	-1.520469	1.54E-37	3.646335	4.189208	2.902257	0.809289	0.674122	0.807462
TSC22D1-AS	-1.041004	0.001958	1.913566	1.124175	1.262951	0.653059	0.356137	0.618528
C1S	-1.007496	6.04E-98	66.76514	64.65735	66.20419	30.38495	31.37369	29.57362
PDE9A	-1.143377	3.50E-11	7.28456	7.810915	6.50212	2.986775	2.865351	3.793313
CNTNAP1	-1.154644	1.87E-55	23.4084	27.85601	24.28214	10.4978	9.993046	11.30564
GOLGA8A	-1.588757	5.09E-61	12.68577	12.34619	10.41088	3.521803	3.084047	3.781652
LINC01615	-1.077694	0.004594	3.449281	2.788454	1.735171	1.314618	0.943208	0.932237
PRG4	-1.082843	2.16E-20	5.567024	5.794394	6.24743	2.495704	2.615458	2.287509

LINC00944	-1.357499	1.27E-08	7.178883	7.776543	8.006966	3.590157	1.818884	2.756572
DDX60	-1.633214	4.14E-17	2.229323	2.604394	2.309499	0.86157	0.73696	0.580693
AC009053.1	-1.203286	0.001837	1.380698	1.841311	1.769464	0.330443	0.54385	0.930523
PKD1P6	-1.150405	1.37E-08	7.03605	6.190268	4.828073	2.56557	2.995269	2.60659
AC138392.1	-1.183535	8.91E-21	25.16539	29.73901	27.98247	10.74236	12.07911	11.99225
CCDC57	-1.086305	6.47E-14	8.616041	8.920518	8.583301	4.808657	6.368542	3.215508
SH3BP5-AS1	-1.280021	0.009991	4.123406	2.508683	3.245999	1.792336	1.297695	1.536823
MATR3	-12.87995	2.99E-14	21.51994	2.601721	30.20624	0.000857	0	0
IFI44L	-1.696349	2.03E-50	8.282567	8.002591	8.322689	1.995564	2.460236	2.641598
TWF1P1	-1.405984	0.000132	3.710658	2.874393	3.613472	1.52278	1.450681	0.681962
HSF4	-1.156991	4.26E-06	4.938267	4.413867	4.947498	2.213269	1.628307	2.533648
ACP7	-1.241427	7.21E-05	2.2937	2.811322	2.871536	1.675587	0.65098	0.782124
NOTCH2NL(	-1.419906	0.001182	0.629292	2.701829	1.052442	0.582087	0.191322	1.052282
HERC2P2	-1.658581	6.29E-74	9.541105	9.904538	8.906081	2.691425	2.599517	2.557306
ADAMTS15	-1.679994	1.44E-35	4.254077	4.017852	3.90846	1.291721	1.240157	1.084372
OAS3	-1.445158	3.06E-22	4.900688	4.355315	4.39866	2.225762	1.516138	2.094079
TNFSF10	-1.172985	0.008995	0.761709	1.484102	1.160012	0.588603	0.472238	0.36526
RPS11P5	-1.133498	0.002243	5.292774	5.323661	4.89349	2.65687	1.66787	2.33813
ITGA4	-1.065371	1.69E-32	8.59103	10.22574	8.889431	4.107532	4.333888	4.153203
STARD4-AS1	-1.846909	3.12E-11	6.048561	3.332992	1.538851	0.778435	1.035372	0.781714
CHRDL1	-1.272373	6.87E-185	79.85338	85.57259	81.67447	33.31504	32.75237	31.23233
GRAMD1C	-1.141741	0.000914	0.948208	1.858978	1.721623	0.337106	0.550138	0.536149
RAB27B	-1.275102	2.75E-57	13.78029	14.23066	12.6233	4.582468	4.755726	5.519207
SPP1	-2.713277	8.11E-97	21.74036	19.47142	19.216	3.200808	2.599247	2.96578
HEPH	-1.323405	2.43E-81	23.35594	23.52814	23.60033	8.878911	7.920157	8.214958
ACAN	-2.292406	3.49E-42	3.56279	3.223473	2.860971	0.634681	0.785598	0.541647
TMEM132B	-1.540028	2.40E-21	2.327992	1.877836	1.707574	0.767714	0.628674	0.572953
SCHIP1	-3.447025	2.22E-28	7.014053	5.972177	4.917637	0.089622	0.268878	0.147408
POM121L9P	-2.035837	5.80E-33	3.832545	3.195252	3.142023	1.012111	0.679615	0.73603
TCAF1P1	-1.983724	2.20E-22	6.431324	6.476717	6.021304	1.98425	1.212175	1.341689
ZFP62	-1.066771	3.20E-11	4.074002	6.460501	3.982925	3.143139	1.880374	1.550279
AL031282.1	-1.05494	1.49E-05	4.087757	2.975044	3.541332	1.398672	1.585251	1.38413
CICP14	-2.075148	1.79E-09	1.598576	1.217905	1.324162	0.274289	0.271304	0.383623
HOXA6	-1.451515	0.00562	1.247268	1.814353	1.611703	0.773732	0.586643	0.284153
ZFHX2-AS1	-1.622797	1.77E-05	1.510093	1.227008	1.455785	0.290319	0.3738	0.630016
PDXDC2P-NI	-1.387087	1.98E-19	5.842941	5.901438	5.448857	1.580176	2.131543	1.817634
HERC2P3	-1.253823	5.68E-05	2.017387	1.535154	1.483377	0.561949	0.299287	0.286516
CADPS2	-1.684897	1.33E-11	1.738443	1.438888	1.229009	0.277247	0.340121	0.536003
RPL37P2	-1.115762	0.006529	7.43542	8.208483	9.957882	5.55326	2.738909	2.912222
FP671120.6	-1.18208	4.66E-05	990.4422	1143.496	1800.826	610.2422	530.5659	514.3701
NAA16	-1.243872	2.52E-09	2.788757	2.005052	2.509457	0.970815	1.00122	0.932571
GEM	-1.02955	1.25E-12	10.02296	12.5008	11.12849	4.838849	4.708026	6.095151
AKR1B10	-1.243712	8.25E-05	3.602906	3.177781	3.759201	1.366081	2.04844	0.972086
BMS1P17	-2.82036	0.000181	1.09792	1.260039	1.201945	0.187136	0.115791	0.138374
AC008522.1	-1.769666	1.56E-10	1.901543	1.621243	1.869831	0.56225	0.465829	0.447057
LINC00476	-1.056892	0.013259	1.600575	1.609175	1.466658	1.131336	0.301575	0.508834
C7	-1.82481	1.17E-42	4.292005	4.644348	4.477863	1.187694	1.556753	1.309886
INMT	-1.410033	4.64E-07	2.135854	3.01175	2.186031	0.942763	1.031224	0.617259
THBS2	-1.18989	2.31E-231	79.92642	80.63416	76.94801	31.6221	33.15218	33.93996
NBPF19	-14.06822	3.51E-06	0.00767	10.87059	7.331607	0	0	0
SPTLC3	-1.373988	1.31E-17	2.781066	2.430275	2.731801	0.888582	1.017567	0.911331
SLC2A5	-1.050523	2.23E-18	7.488764	6.332347	6.834683	2.980707	3.372053	2.951004
GOLGA8B	-1.613525	1.34E-59	7.588965	8.2066	8.129845	3.363453	3.077205	2.508058
ICAM1	-1.464727	4.87E-67	22.8694	24.81865	21.05095	7.959184	7.813704	7.936109
AL121772.3	-2.114016	0.014746	1.361911	1.189388	1.157206	0	0.430578	0.365997
PKD1P3	-1.743907	1.01E-52	6.44242	5.342111	5.331155	1.542961	1.697455	1.608497
APOL6	-1.061745	1.25E-24	5.199592	5.006223	4.646428	1.977377	2.221937	2.58164
NDP	-1.176331	9.03E-14	10.48149	8.19395	9.620765	3.562503	4.2413	4.293368

URAHP	-1.490682	0.01017	0.753486	0.900786	1.50456	0.281943	0.600612	0.200087
SLC25A27	-1.456655	4.16E-05	2.02392	1.728874	1.902557	0.598502	0.67676	0.334066
LINC01881	-1.200138	0.00319	2.766297	2.827105	1.571649	0.758382	1.215317	0.936841
SUCLG2-AS1	-1.092447	0.00625	1.860236	1.50704	0.604308	0.583408	0.739058	0.474715
NETO2	-1.039693	3.69E-212	107.2095	101.624	97.68205	44.09134	41.22118	48.5941
ARHGAP28	-1.17946	7.60E-05	1.162994	1.504494	1.061761	0.539107	0.297533	0.728567
SNED1	-1.682923	1.39E-195	21.84461	24.0909	22.90897	7.802725	7.117867	6.6759
AC004263.2	-2.5395	1.07E-12	1.655581	1.485651	1.668379	0.222114	0.31823	0.233874
VIM-AS1	-3.286122	0.005786	26.31227	124.2058	2.670853	3.806982	4.516008	3.947224
NCAM2	-1.12901	3.88E-07	1.474455	1.834634	1.471592	0.497641	0.516478	0.869254
GSAP	-1.016167	4.25E-11	6.315519	5.025555	6.38154	2.854072	3.242786	2.8049
MYLIP	-1.205319	3.50E-09	2.537359	3.019315	3.203917	1.145101	1.179887	1.296514
PTGS1	-1.010081	2.08E-26	9.969736	9.466087	9.410609	4.384097	5.359414	4.467984
NPIPB14P	-1.705388	0.000989	1.068393	1.592765	2.191239	0.382993	0.556786	0.579903
CCDC102B	-1.288506	1.39E-07	3.044873	4.229696	4.049054	1.962152	1.487775	1.348108
LY75	-2.01929	4.07E-11	1.022687	0.78166	1.251943	0.243958	0.125852	0.351673
AC022384.1	-3.07693	8.85E-07	0.517527	2.024071	0.987294	0.100161	0.038425	0.300357
AP006623.1	-1.025666	0.016071	0.994725	1.205355	0.998176	0.3011	0.45739	0.725458
MXRA5	-1.181867	6.57E-14	1.471899	1.393915	1.310332	0.607509	0.579876	0.566027
AC159540.2	-1.029021	4.59E-07	3.189069	2.870376	3.144767	1.073288	1.552311	1.785736
AC079298.3	-1.781079	3.37E-22	6.349975	6.156616	8.306534	2.47389	2.576052	2.475638
SCD	-1.861716	0	87.18168	84.95033	83.79737	21.48618	21.66033	23.97346
AKR1C3	-1.091511	1.65E-57	72.90846	69.43465	67.41859	29.97356	31.02908	32.65722
ARRDC4	-1.007507	3.31E-151	75.56601	74.10794	71.95905	34.58143	35.89357	34.5892
LAMA2	-1.148518	5.77E-26	4.654256	4.50903	4.247859	2.111073	2.013542	1.635571
RSKR	-1.207097	0.001143	1.361627	1.859586	1.417018	0.868383	0.529453	0.425877
AL356515.1	-3.087209	0.010871	1.087564	1.290236	1.249165	0	0.391447	0
AL732372.2	-1.62896	0.00225	2.364943	1.983864	1.930509	0.37525	0.577126	0.663142
EIF4BP6	-1.091736	0.000424	2.420167	2.041047	3.064151	0.922775	0.944244	1.501187
SOHLH2	-1.561036	0.000264	1.36459	1.262586	0.856156	0.376707	0.50562	0.238262
MT-TY	-1.019731	3.91E-173	4642.016	4703.992	4638.441	2210.244	2184.952	2178.55
SLC6A6	-1.118987	2.08E-48	10.06578	10.09306	9.300053	4.772617	4.489254	4.764011
RAPH1	-1.226852	1.76E-21	4.264249	6.717338	4.056131	2.384488	1.531858	2.658591
AC018665.1	-1.250323	0.005829	1.747767	1.277516	1.226203	0.721282	0.333393	0.64142
STMN2	-1.008058	1.73E-28	31.31091	34.44612	39.22556	15.74694	17.09925	16.19559
AC079949.2	-1.340984	2.80E-12	4.412098	4.095181	3.404969	1.319552	1.710519	1.443816
SETD5	-1.200077	4.74E-31	15.49973	15.11028	16.20785	6.815456	7.41056	6.886571
PARGP1	-1.579307	0.001807	0.916163	1.174966	1.343211	0.358331	0.486564	0.515863
CCL20	-1.043207	4.34E-08	13.47241	13.96061	14.05101	6.022859	5.714935	8.409226
ZNF169	-1.655775	0.001681	1.537323	1.309097	1.306514	0.386606	0.075941	0.697275
TBC1D3D	-1.397773	0.000519	4.876391	2.196458	1.347177	1.218517	1.159401	0.642742
CPA6	-1.767175	0.000443	1.250372	1.024282	0.783606	0.118488	0.383502	0.348407
